# Transcriptional Reprogramming Differentiates Active from Inactive ESR1 Fusions in Endocrine Therapy-Refractory Metastatic Breast Cancer

**DOI:** 10.1158/0008-5472.CAN-21-1256

**Published:** 2021-10-28

**Authors:** Xuxu Gou, Meenakshi Anurag, Jonathan T. Lei, Beom-Jun Kim, Purba Singh, Sinem Seker, Diana Fandino, Airi Han, Saif Rehman, Jianhong Hu, Viktoriya Korchina, Harshavardhan Doddapaneni, Lacey E. Dobrolecki, Nicholas Mitsiades, Michael T. Lewis, Alana L. Welm, Shunqiang Li, Adrian V. Lee, Dan R. Robinson, Charles E. Foulds, Matthew J. Ellis

**Affiliations:** 1 Lester and Sue Smith Breast Center, Baylor College of Medicine, Houston, Texas.; 2 Graduate Program in Translational Biology and Molecular Medicine, Baylor College of Medicine, Houston, Texas.; 3 Department of Medicine, Baylor College of Medicine, Houston, Texas.; 4 Department of Human and Molecular Genetics, Baylor College of Medicine, Houston, Texas.; 5 Department of Surgery, Yonsei University Wonju College of Medicine, Wonju, Korea.; 6 Queens' College, University of Cambridge, Cambridge, UK.; 7 Human Genome Sequencing Center, Baylor College of Medicine, Houston, Texas.; 8 Department of Molecular and Cellular Biology, Baylor College of Medicine, Houston, Texas.; 9 Dan L. Duncan Cancer Center, Baylor College of Medicine, Houston, Texas.; 10 Department of Radiology, Baylor College of Medicine, Houston, Texas.; 11 Department of Oncological Sciences, Huntsman Cancer Institute, University of Utah, Salt Lake City, Utah.; 12 Division of Oncology, Department of Internal Medicine, Washington University School of Medicine, Saint Louis, Missouri.; 13 Department of Pharmacology and Chemical Biology, UPMC Hillman Cancer Center, University of Pittsburgh, Pittsburgh, Pennsylvania.; 14 Women's Cancer Research Center, Magee-Women's Research Institute, Pittsburgh, Pennsylvania.; 15 Michigan Center for Translational Pathology, University of Michigan, Ann Arbor, Michigan.; 16 Department of Pathology, University of Michigan, Ann Arbor, Michigan.

## Abstract

Genomic analysis has recently identified multiple *ESR1* gene translocations in estrogen receptor alpha–positive (ERα^+^) metastatic breast cancer (MBC) that encode chimeric proteins whereby the ESR1 ligand binding domain (LBD) is replaced by C-terminal sequences from many different gene partners. Here we functionally screened 15 ESR1 fusions and identified 10 that promoted estradiol-independent cell growth, motility, invasion, epithelial-to-mesenchymal transition, and resistance to fulvestrant. RNA sequencing identified a gene expression pattern specific to functionally active ESR1 gene fusions that was subsequently reduced to a diagnostic 24-gene signature. This signature was further examined in 20 ERα^+^ patient-derived xenografts and in 55 ERα^+^ MBC samples. The 24-gene signature successfully identified cases harboring *ESR1* gene fusions and also accurately diagnosed the presence of activating *ESR1* LBD point mutations. Therefore, the 24-gene signature represents an efficient approach to screening samples for the presence of diverse somatic *ESR1* mutations and translocations that drive endocrine treatment failure in MBC.

**Significance:**

This study identifies a gene signature diagnostic for functional ESR1 fusions that drive poor outcome in advanced breast cancer, which could also help guide precision medicine approaches in patients harboring *ESR1* mutations.

## Introduction

The majority of breast cancers (∼70%) are initially diagnosed as estrogen receptor-alpha positive (ERα^+^) and are dependent on 17β estradiol (E2) for growth (1). Thus, endocrine therapies (ET) either induce estrogen deprivation, for example, through aromatase inhibition (AI), or directly target the ERα ligand binding domain (LBD) with selective ER modulation (e.g., tamoxifen) or degradation (e.g., fulvestrant; ref. 1). However, acquired ET resistance is common, and is often associated with somatic mutations in the gene encoding ERα, *ESR1*. The most extensively studied examples are point mutations in the LBD that result in ERα proteins with ligand-independent activity. Common examples include Y537S and D538G (2, 3). These mutations typically arise after patients have undergone extensive endocrine treatment and can be present in up to 40% of patients with ERα^+^ metastatic breast cancer (MBC; refs. 4, 5).

Emerging evidence indicates that chromosomal translocations involving the *ESR1* gene can also drive ET resistance through the formation of chimeric transcription factors (TF) with constitutive activity (6, 7). The first described example was an ESR1-e6>YAP1 fusion detected by whole-genome sequencing and RNA sequencing (RNA-seq) in samples from a patient with rapid onset ET resistance (6). The fusion protein was encoded by an interchromosomal translocation event that brought *ESR1* exons 1 to 6 (ESR1-e6) on chromosome (chr) 6q into the *YAP1* locus on chr11q, thereby replacing the entire LBD with transactivation domain (TAD) sequences from this Hippo pathway transcriptional coactivator (CoA). A patient-derived xenograft (PDX) established from the patient's tumor (WHIM18) also exhibited ET resistance. We subsequently identified another in-frame exon 6 fusion, ESR1-e6>PCDH11X in a male patient with ER^+^ MBC as a result of a chr6q>Xq translocation. This second example was a harbinger of complexity to come, because *PCDH11X* encodes a protocadherin without known transcriptional functions. Both fusions not only induced ET-resistant tumor growth but also increased lung metastasis in xenograft mice models (7). Another in-frame ESR1-e6 fusion, ESR1-e6>NOP2, was detected in a primary tumor but was found to be transcriptionally inactive (7). This example suggested that the mere presence of an ESR1-e6 fusion that generates a stable chimeric protein is insufficient evidence that an ET-resistance driver has been identified.

Multiple additional ESR1-e6 fusions have now been identified from ER^+^ MBC patients. In a study by Lee and colleagues, three ESR1-e6 fusions (ESR1-e6>DAB2, ESR1-e6>GYG1, and ESR1-e6>SOX9) were shown to activate an estrogen response element (ERE)-driven luciferase reporter construct in transfected HEK293 cells (8). In the MET500 study (9), three in-frame fusions (ESR1-e6>ARNT2-e18, ESR1-e6>PCMT1, and ESR1-e6>ARID1B) were identified in samples from MBC patients and were provided to us for functional studies in a pre-publication personal communication. To further investigate the significance of in-frame ESR1-e6 fusion genes, each example was screened for ET-resistance induction *in vitro*, defined as E2-independent and fulvestrant-resistant growth and increased motility, in two ER^+^ breast cancer cell lines (T47D and MCF7). RNA-seq was undertaken to understand the transcriptional reprogramming induced by active ESR1 fusions. We subsequently trained a 24-gene signature that was characteristic of the presence of an active ESR1 fusion as compared with an inactive fusion or wild-type (WT) ESR1. In the effort to validate this signature in ER^+^ PDXs and in clinical samples, activating LBD point mutations were discovered to also induce this gene expression signature. These data suggest that despite the remarkable diversity of mutations in the *ESR1* gene, these somatic events converge on a common pathogenic transcriptional reprogramming mechanism to drive poor outcome and ET resistance in MBC.

## Materials and Methods

### Cell culture

Growth conditions for T47D (ATCC; cat #HTB-133, RRID:CVCL_0553) and MCF7 (ATCC; cat #HTB-22, RRID:CVCL_0031) cells are described in Lei and colleagues (7) and detailed in Supplementary Information. ERα ligands (E2 and fulvestrant) were purchased from Sigma (E4389) and Selleckchem (S1191), respectively.

### Subcloning of ESR1 mutants into a lentiviral expression vector

Lentiviral vectors expressing C-terminal HA-tagged yellow fluorescent protein (YFP), truncated ESR1-e6, ESR1-WT, ESR1-Y537S, ESR1-D538G, ESR1-e6>YAP1, and ESR1-e6>PCDH11X were previously described (6, 7). HA-tagged cDNAs encoding the new ESR1-e6 fusions in this study were constructed in a similar fashion as detailed in Supplementary Information.

### Generation of lentiviral stable ESR1-mutant expressing cell lines

Lentivirus were produced as described (6) by cotransfecting the above *ESR1* cDNA lentiviral vectors with the packaging plasmids pMD2.G (RRID:Addgene_12259) and psPAX2 (RRID:Addgene_12260) into HEK293T (ATCC; cat #CRL-3216, RRID:CVCL_0063) cells using Lipofectamine 2000 (Invitrogen, cat #11668-027). Transduced breast cancer cells were selected with 2 μg/mL puromycin (Sigma; cat #P8833) for 7 days. Expression of various ESR1 proteins was validated using immunoblotting.

### Immunoblotting and immunoprecipitation

Cells were harvested and whole-cell lysates were prepared in RIPA lysis buffer as described (7) or in MIB lysis buffer (10) supplemented with 1× protease inhibitors and 1× phosphatase inhibitors (Roche) by sonication for 2 minutes. To make ER^+^ PDX tumor lysates, frozen PDX tumors were cryopulverized with a Covaris CP02 Pulverizer, and then protein was extracted in MIB lysis buffer with sonication. Protein concentration determination and SDS-PAGE (20 μg protein per lane) were performed as described (7). Immunoblotting of nitrocellulose membranes was performed as described (7). Primary and HRP-conjugated secondary antibodies used are listed in the Supplementary Information.

Immunoprecipitation was performed as described (7), using 2 mg of lysates from hormone-deprived T47D cells with or without E2 treatment (100 nmol/L for 45 minutes). Lysates were incubated with 2 μg anti-HA tag antibody (Santa Cruz Biotechnology; cat #sc-7392, RRID:AB_627809) or mouse IgG (Cell Signaling Technology; cat #61656, RRID:AB_2799613) control, followed by capture of antibody–antigen complexes with protein A magnetic beads (Bio-Rad, cat #1614013) as described (7). Immunoprecipitated proteins, as well as 20 μg of whole-cell lysates (1% inputs), were analyzed by immunoblotting.

### Cell growth, motility, and invasion assays

Cell growth assays of different ESR1 fusion protein expressing breast cancer cells that were first hormone-deprived and then subsequently treated either with 100 nmol/L fulvestrant in the presence or absence of 10 nmol/L E2 for 7 to 10 days were performed in 96-well plates using an alamarBlue assay as described (7). Cell growth reading values were normalized to that of control YFP cells, –E2.

Cell motility was detected using a scratch wound assay of hormone-deprived stable cells in a 96-well ImageLock plate (Essen BioScience) that were pretreated for 2 hours with mitomycin C (50 ng/mL for T47D and 200 ng/mL for MCF7; Sigma, M4287) before wounding as described (7). Wound images were acquired every 6 hours for 72 hours by an IncuCyte camera (Essen Bioscience) in a cell culture incubator. Relative wound densities (RWD) were calculated as density in the wound area relative to that outside the wound area to account for confounding proliferation.

The cell invasion assay was performed and analyzed in a similar manner to the scratch wound assay except that cells were plated on Matrigel-coated plate. After the scratch was generated on cell monolayer, 50 μL Matrigel solution was added to the wells, thus filling the scratch region and 100 μL of additional culture media containing mitomycin C.

### RNA-seq and analysis

Different *ESR1* cDNA stably expressing T47D cell lines were cultured in CSS media for 5 days followed by treatment with or without 10 nmol/L E2 for 2 days. RNA was isolated using the RNeasy Mini Kit (QIAGEN; cat #74106) and treated with DNase (QIAGEN; cat #79254) to remove genomic DNA. The Genomic and RNA Profiling (GARP) Core at BCM confirmed concentration (using a NanoDrop spectrophotometer) and integrity (using an Agilent Bioanalyzer). The GARP core then made mRNA libraries and performed sequencing on an Illumina NovaSeq 6000 sequencing instrument as described in detail in Supplementary Information. For RNA-seq on isolated ER^+^ PDX tumors, frozen PDX tumors were cryopulverized as above and total RNA was isolated using the RNeasy kit. RNA-seq was performed at the Human Genome Sequencing Center at BCM as described in detail in Supplementary Information.

For RNA-seq analysis, paired-end 150 bp reads were aligned to the hg19 (GRCh37) reference genome using RSEM v1.2.31 (RSEM, RRID:SCR_013027; ref. 11) and Bowtie 2 (12). Transcripts per million values calculated by RSEM were log_2_ transformed and subjected to heatmap generation using Morpheus (https://software.broadinstitute.org/morpheus; Morpheus, RRID:SCR_014975). Unsupervised hierarchical clustering and identification of differentially expressed genes in active ESR1 fusion protein expressing cells to cells expressing inactive fusions and controls are described in Supplementary Information.

### Whole-exome sequencing and analysis

DNA was isolated from the ER^+^ PDX tumors using a QIAamp DNA Mini Kit (QIAGEN; cat #51304). WES data were generated by the Human Genome Sequencing Center at BCM using the Illumina platform as described in detail in Supplementary Information. Tools used for somatic *ESR1* LBD gene variant calling were Strelka2, Mutect2, and CARNAC (v 0.2.5b9) as described in Supplementary Information. *ESR1-e6>YAP1* fusion was detected in WHIM18 previously (6).

### Reverse transcription–quantitative PCR

RNA was isolated from hormone-deprived stable T47D cells as above with concentration determined using a NanoDrop spectrophotometer. One-step RT-qPCR was conducted using 50 ng RNA incubated with SsoAdvanced Universal SYBR Green Supermix (Bio-Rad; cat #1725274), iScript reverse transcriptase (Bio-Rad; cat #170-8891) and 0.5 μm primers (Sigma) as described (7). All samples were run in triplicate on a CFX96 thermal cycler (Bio-Rad).

### Immunofluorescence

Immunofluorescence was performed of different HA-tagged ESR1 fusion proteins expressed in hormone-deprived T47D cells as described (7). These proteins were detected using an anti-HA antibody (Cell Signaling Technology; cat #2367, RRID:AB_10691311, 1:50) and goat anti-mouse IgG secondary antibody (Alexa Fluor 568, Molecular Probes; cat #A-11004, RRID:AB_2534072, 1:1,000) as described in Supplementary Information. Nuclei were detected by DAPI staining as described (7).

### PDX models

The PDX models were previously described (6, 13, 14). All animal procedures were approved by the Institutional Animal Care and Use Committee at BCM (protocol #AN-6934). Two- to 3-mm pieces from PDX tumors were engrafted into cleared mammary fat pads of 3- to 4-week-old ovariectomized SCID/beige mice (Charles River). Mice were randomized to receive sterile drinking water with or without 8 μg/mL E2 supplementation (*n* = 7–16 per PDX line per arm). Tumor volumes were measured by caliper every 3–4 days, and were calculated by *V* = 4/3 × π × (width/2)2 × (length/2). Mice were sacrificed when tumors reached 1.5 cm^3^ or at the study endpoint. Tumors were harvested and frozen in liquid nitrogen for storage. Additional information on BCM and HCI PDX models is available at pdxportal.research.bcm.edu/.

### Gene signature and ROC curve analysis

The signature performance was calculated as follows: Accuracy = (TP + TN)/(TP + TN + FP + FN), sensitivity = TP/(TP + FN), specificity = TN/(TN + FP), in which TP, true positive; TN, true negative; FP, false positive; FN, false negative. Receiver operating characteristic (ROC) curve analysis was performed using “pROC” package in R (15).

### ERE DNA pulldown assays

These assays were modified from the established protocol of HeLa cell nuclear extract (NE) supplemented with recombinant estrogen receptors (16, 17). Briefly, nuclear extracts were made from T47D cell lines expressing YFP or different ESR1 fusion proteins (15–25 15-cm dishes used) exactly as published (18). Pulldown assays used 1 mg of T47D cell NE to resuspend 60 μL Dynabeads M-280 Streptavidin that was prebound to 3 μg biotinylated 4xERE-E4 921 bp DNA. Incubation occurred at 4°C with gentle rotation for roughly 2 hours, followed by pelleting beads with a magnetic rack and quick washes as described (16, 17). Final beads were resuspended in 30 μL 2xSDS-sample buffer, boiled, and 30% of the final supernatants were loaded onto 4% to 15% gradient SDS-PAGE gels. After transfer to nitrocellulose, immunoblots were probed with N-terminal ERα (Millipore; cat #04-820, RRID:AB_1587018) or DNA-PKcs (Santa Cruz Biotechnology; cat #sc-5282, RRID:AB_2172848) antibodies with ECL-based signal detection on a Bio-Rad Imaging System.

### Statistical tests and analyses of publicly available data

**Figure 1.**
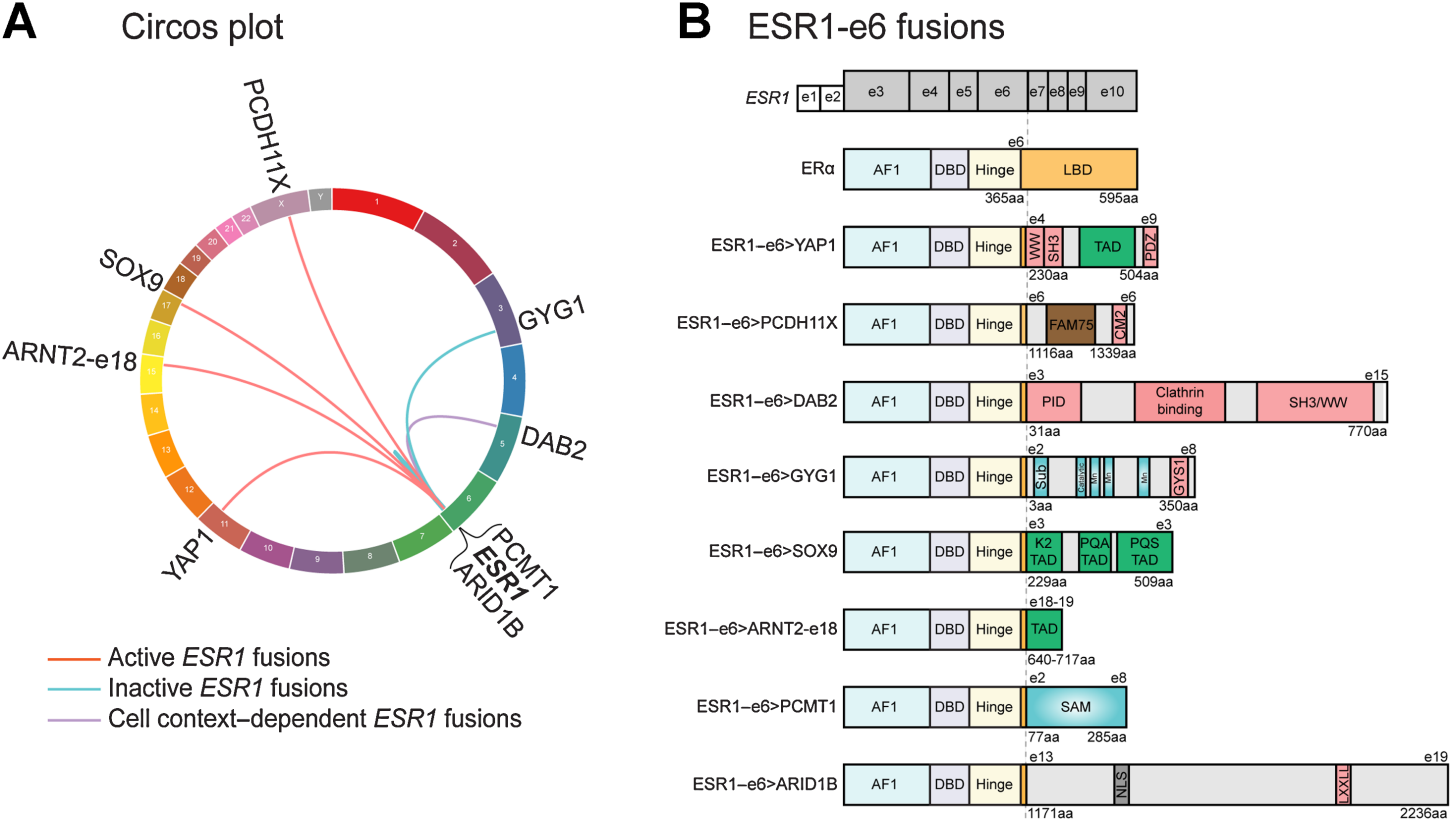
In-frame ESR1-e6 fusions identified in ER^+^ MBC patients. **A,** Circos plot depicting *ESR1* fusion events identified from ER^+^ MBC patients. The *ESR1* gene is connected to its 3′ partner genes with lines. **B,** In-frame *ESR1* fusions in ER^+^ MBC possess a common structure whereby the first 6 exons (two untranslated exons and four coding exons in gray, exons 3–6) of *ESR1* fuse in-frame to C-terminal sequences from partner genes. Key for domains in the WT ERα protein: AF1, activation function 1 domain; DBD, DNA-binding domain; Hinge, domain connecting DBD and LBD; and LBD, ligand-binding domain. Pink boxes in partner proteins mediate protein-protein interactions, including WW binding motifs, SH3 binding motifs, a PDZ domain, a conserved motif 2 (CM2), a phospho-tyrosine interaction domain (PID), an interaction with glycogen synthase 1 region (GYS1), and an LXXLL motif. Green boxes represent known transcriptional activation domains (TAD). The brown box represents the FAM75 domain of unknown function. Blue domains have enzymatic activities, including substrate binding site (Sub), catalytic site, three manganese binding sites (Mn), and an S-adenosylmethionine-dependent methyltransferase domain (SAM). The gray box labeled NLS represents a nuclear localization signal.

Unpaired two-tailed Student *t* test and ANOVA were performed with GraphPad Prism 9 (GraphPad Prism, RRID:SCR_002798), as indicated in the figure legends. *P* values less than 0.05 were considered statistically significant. The protein domains with functional information in Figs. 1B and 5A were extracted from UniProt Knowledgebase (19). RNA-seq data derived from ER^+^ MBC along with *ESR1* mutation status (*n* = 55) were downloaded from the MET500 web portal (https://met500.path.med.umich.edu/). Cases with an *ESR1* mRNA expression >1 FPKM were considered as ER^+^, using a previously described criterion (20).

### Data availability

RNA-seq data from T47D cells and WES and RNA-seq data from ER^+^ PDX tumors in this study are being submitted to Gene Expression Omnibus (GEO). Until an accession number is provided, these raw data are available upon request from the corresponding author.

A full description of all methods and reagents can be found in Supplementary Information.

## Results

### A subset of in-frame ESR1-e6 fusions identified in ER^+^ MBC patients drives ET-resistant growth and promotes hormone-independent motility and invasion of ER^+^ breast cancer cells

We initially studied six newly identified in-frame *ESR1* fusions detected in samples from MBC patients and compared them to the ESR1-e6>YAP1 and ESR1-e6>PCDH11X examples we described previously (7). Some fusion examples arose from interchromosomal translocations, such as ESR1-e6>DAB2, ESR1-e6>GYG1, ESR1-e6>SOX9 (Lee Laboratory; ref. 8), and ESR1-e6>ARNT2-e18 (Robinson, D. personal communication). Two other fusions were formed by rearrangements within chromosome 6, ESR1-e6>PCMT1 and ESR1-e6>ARID1B (D. Robinson, personal communication; Fig. 1A). All six examples followed a structure established by the original ESR1-e6>YAP1 fusion whereby the first six exons of *ESR1* were fused in-frame to C-terminal partner genes, completely replacing the ERα LBD with an alternative C-terminus. We noted two classes functionally, (i) TF and transcription coactivator (CoA) fusions or (ii) fusions with genes without previously established (direct) functions in gene transcription (Fig. 1B).

**Figure 2.**
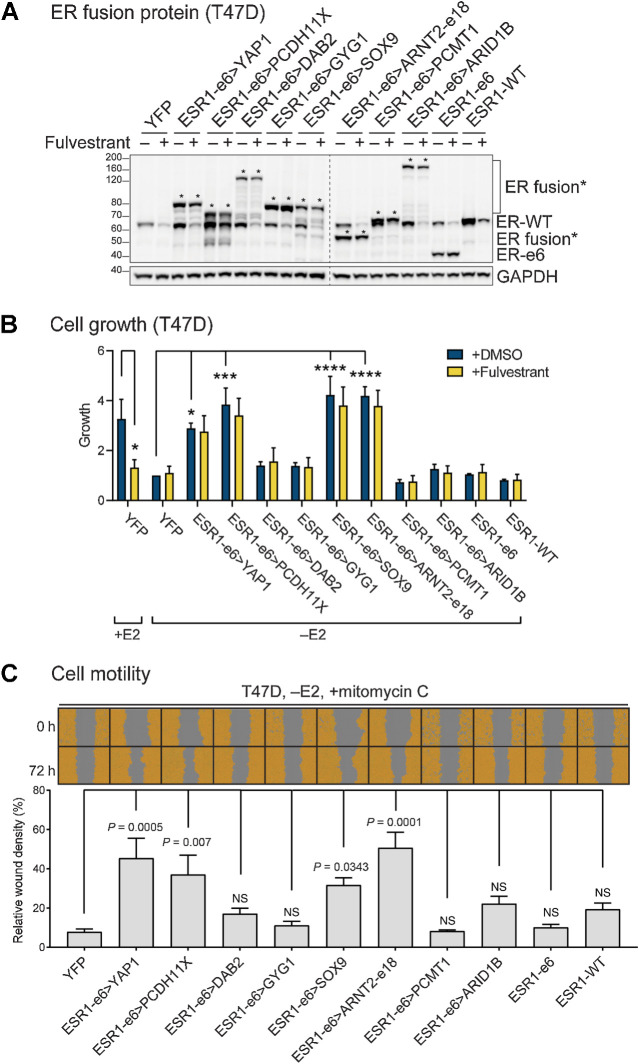
ESR1 fusion proteins drive ET-resistant growth and promote hormone-independent motility of ER^+^ breast cancer cells. **A,** Immunoblotting of ERα and ESR1 fusion proteins with an N-terminal ERα antibody in lysates made from hormone-deprived stable T47D cells. Asterisks indicate ER fusion proteins. GAPDH serves as a loading control. The dashed line indicates two separate blots that were conducted at the same time. The representative image is from three independent experiments. **B,** Cell growth was assayed in hormone-deprived stable cells (mean ± SEM; *n* = 3). One-way ANOVA followed by Dunnett multiple comparisons test was used to compare data of hormone-deprived ESR1 fusion expressing cells with YFP control cells in the vehicle (+DMSO) group. Two-way ANOVA followed by Bonferroni test was used for multiple comparisons for each stable cell line after 100 nmol/L fulvestrant treatment in the presence or absence of 10 nmol/L estradiol (E2). *, *P* < 0.05; ***, *P* < 0.001; ****, *P* < 0.0001. See Supplementary Fig. S1B for the complete data. **C,** Cell motility was detected using scratch wound assays in hormone-deprived stable T47D cells, treated with mitomycin C to block proliferation (mean ± SEM; *n* = 3). Cells are pseudo-colored orange to aid visualization. One-way ANOVA followed by Dunnett multiple comparisons test was used to compare each stable T47D cell line with YFP control cells. NS, not significant.

To characterize each chimeric ESR1 fusion protein, HA-tagged cDNA constructs were expressed in two ER^+^ breast cancer cell lines (T47D and MCF7) by lentiviral transduction. Stable cell lines expressing YFP were generated as negative controls. Truncated *ESR1* (ESR1-e6 protein) and WT *ESR1* (ESR1-WT protein) were also stably expressed to provide overexpression controls (Fig. 2A; Supplementary Fig. S1A). When cells were treated with 100 nmol/L fulvestrant, a selective ERα degrader that inhibits endogenous ERα (21), the level of ESR1 fusion protein was predictably unaffected. In comparison, the WT ERα protein was reliably degraded, providing an endogenous control for fulvestrant activity (Fig. 2A; Supplementary Fig. S1A). To investigate whether ESR1 fusion proteins drove ET resistance, cell lines expressing *ESR1* fusion cDNAs were hormone-deprived for 7 days in charcoal stripped serum-containing phenol red-free, RPMI media (CSS media) and then treated for 7 to 10 days with or without 10 nmol/L E2 and with or without 100 nmol/L fulvestrant. Cell growth was measured using an alamarBlue assay. Similar to ESR1-e6>YAP1 and ESR1-e6>PCDH11X (7), ESR1-e6>SOX9 and ESR1-e6>ARNT2-e18 conferred E2-independent growth of T47D cells compared with the YFP controls (–E2, +DMSO; Fig. 2B, all four conditions are shown in Supplementary Fig. S1B) in a manner that was uniformly resistant to fulvestrant (Fig. 2B). Although the four other ESR1-e6 fusions studied (ESR1-e6>DAB2, ESR1-e6>GYG1, ESR1-e6>PCMT1, and ESR1-e6>ARID1B) produced stable proteins, they did not promote ET-resistant growth of T47D cells with inactivity resembling the controls (truncated ESR1-e6 protein alone, ESR1-WT and YFP). The *GYG1* example is an important exception, because this is an in-frame, interchromosomal translocation that might have been expected to be active. Although cell growth was induced by E2 treatment regardless of the presence of an ESR1 fusion protein, only ESR1-e6>YAP1, ESR1-e6>PCDH11X, ESR1-e6>SOX9, and ESR1-e6>ARNT2-e18 drove significantly higher growth than YFP control cells in presence of fulvestrant (+E2, +Fulvestrant; Supplementary Fig. S1B). The elevated E2-independent, fulvestrant-resistant growth phenotypes were further validated in MCF7 cells (Supplementary Fig. S1C). Interestingly, ESR1-e6>DAB2 demonstrated E2-independent, fulvestrant-resistant growth in MCF7 cells, but not in T47D cells, suggesting the activity of this fusion was cell line selective.

To determine whether each fusion protein promoted cell motility, as an initial measure of metastasis-driving potential, stable T47D or MCF7 cells were hormone-deprived and pretreated with mitomycin C to inhibit cellular proliferation. Cell monolayers were scratched and wound images were monitored for 72 hours. RWDs were measured as density in the wound area relative to that outside the wound area. All four growth-promoting ESR1 fusion proteins, ESR1-e6>YAP1, ESR1-e6>PCDH11X, ESR1-e6>SOX9, and ESR1-e6>ARNT2-e18, induced higher cell migration than controls in a hormone-independent manner (–E2; Fig. 2C; Supplementary Fig. S1D). Consistent with the proliferation data, ESR1-e6>DAB2 also promoted cell motility in MCF7, but not in T47D cells. Importantly, the expression levels of the functionally active ESR1 fusion proteins were similar to the inactive examples (Fig. 2A; Supplementary Fig. S1A), suggesting that the inactivity of individual ESR1 fusion proteins was not due to differential expression or stability. ESR1-e6>YAP1, ESR1-e6>PCDH11X, ESR1-e6>SOX9, and ESR1-e6>ARNT2-e18 also promoted more invasion through Matrigel than either the controls (YFP, ESR1-e6, and ESR1-WT) or the inactive fusions (Supplementary Fig. S2).

### Active ESR1 fusion proteins upregulate expression of estrogen response genes and EMT genes

**Figure 3.**
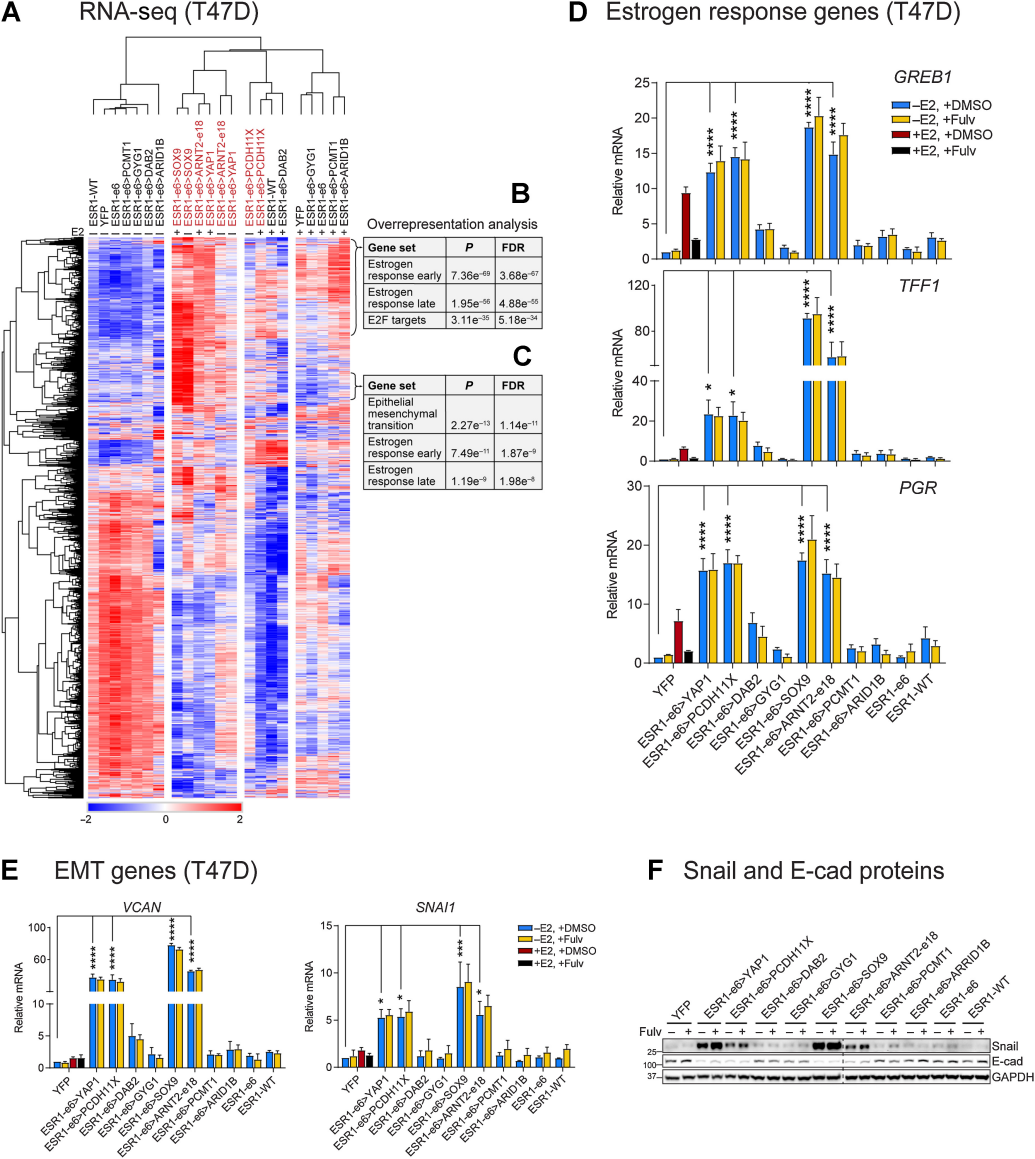
Active ESR1 fusion proteins upregulate expression of estrogen response and EMT genes. **A,** Heatmap showing the differently expressed genes in the T47D RNA-seq data. **B** and **C,** Active ESR1 fusions upregulate expression of two clusters of estrogen response (early and late) and EMT-related genes as indicated. **D** and **E**, Expression of estrogen response genes (*GREB1*, *TFF1*, and *PGR*) and EMT-related genes (*VCAN* and *SNAI1*) was measured by RT-qPCR in E2-deprived T47D cells treated with vehicle (+DMSO) or 100 nmol/L fulvestrant (+Fulv) in the absence (–E2) or presence (+E2) of 10 nmol/L E2 for 48 hours. Values were normalized to *GAPDH* mRNA, and relative expression was calculated as fold change to YFP, –E2 (mean ± SEM; *n* = 3). One-way ANOVA followed by Dunnett multiple comparisons test was used to compare each E2-deprived T47D cell line with YFP control cells (*, *P* < 0.05; ***, *P* < 0.001; ****, *P* < 0.0001). Two-way ANOVA followed by Bonferroni test was used for multiple comparisons for each stable cell after 100 nmol/L fulvestrant treatment. **F,** Snail and E-cadherin proteins were measured by immunoblotting in E2-deprived cells treated with or without 100 nmol/L fulvestrant (Fulv). GAPDH protein served as a loading control. The dashed line indicates two separate blots that were conducted at the same time. The representative image is from three independent experiments.

To define the transcriptional profile driven by active ESR1 fusion proteins, RNA-seq was performed on T47D cells expressing these *ESR1* fusion cDNAs as well as control (YFP, ESR1-e6, and ESR1-WT) cells in the presence and absence of E2. Hierarchical clustering showed that T47D cells expressing ESR1-e6>YAP1, ESR1-e6>PCDH11X, ESR1-e6>SOX9, and ESR1-e6>ARNT2-e18 fusions clustered distinctly from other ESR1-e6 fusions and control cells under E2-deprived conditions (–E2; Fig. 3A). All four active fusions demonstrated an expression pattern similar to control cells treated with E2, consistent with potent hormone-independent transcriptional activation of “estrogen response” genes (Fig. 3B). Interestingly, active ESR1 fusion proteins also upregulated a cluster of genes that were not observed in the control cells stimulated by E2 (Fig. 3C). Overrepresentation analysis revealed a significant enrichment of “estrogen response” pathways and an epithelial-to-mesenchymal transition (EMT) signature specific to active ESR1 fusion proteins and thus consistent with data presented above on invasion and motility (Fig. 3B and C). The expression of three canonical estrogen response genes in stably transfected T47D cells was validated using RT-qPCR. ESR1-e6>YAP1, ESR1-e6>PCDH11X, ESR1-e6>SOX9, and ESR1-e6>ARNT2-e18 significantly induced the expression of *GREB1*, *TFF1*, and *PGR* mRNA, all three known as direct ERα targets (22) (Fig. 3D), in a hormone-independent, fulvestrant-resistant manner compared with YFP controls. These transcriptionally active ESR1 fusion proteins also upregulated two EMT-related genes, *SNAI1* (Snail), encoding a master TF that induces EMT (23) by transcriptional repression of epithelial genes such as *E-cadherin* (24), and *VCAN* (versican; Fig. 3E). The elevated expression of Snail protein and a corresponding decrease of E-cadherin (E-cad) were confirmed by immunoblotting (Fig. 3F). As expected, the expression of these genes were unaffected by fulvestrant treatment. The other ESR1-e6 fusion examples, ESR1-e6>DAB2, ESR1-e6>GYG1, ESR1-e6>PCMT1, and ESR1-e6>ARID1B, did not induce E2-independent activation of ERα target genes and EMT-related genes in T47D cells. The induction of Snail protein was also reproduced in MCF7 cells; however, ESR1-e6>PCDH11X displayed a minor upregulation compared with other transcriptionally active fusions (Supplementary Fig. S1A). Consistent with the observed MCF7 cell line-selective increase in cell growth and migration, ESR1-e6>DAB2 also upregulated Snail expression compared with YFP control and the inactive fusions. Consistent with above T47D cell line data, E-cadherin protein was reduced in MCF7 cells expressing active ESR1 fusion proteins (Supplementary Fig. S1A).

Additional experiments were conducted to demonstrate that inactive ESR1 fusion proteins enter the nucleus as the nuclear translocation signal is preserved. Also, there is no biochemical evidence for heterodimer formation with WT ERα (Supplementary Fig. S3). These data imply that the inactive *ESR1* fusion genes are not dominant negative, a result consistent with the normal E2-induced growth in inactive fusion-expressing cells.

### Active ESR1 fusion proteins induce a characteristic, hormone-independent transcriptional signature

**Figure 4.**
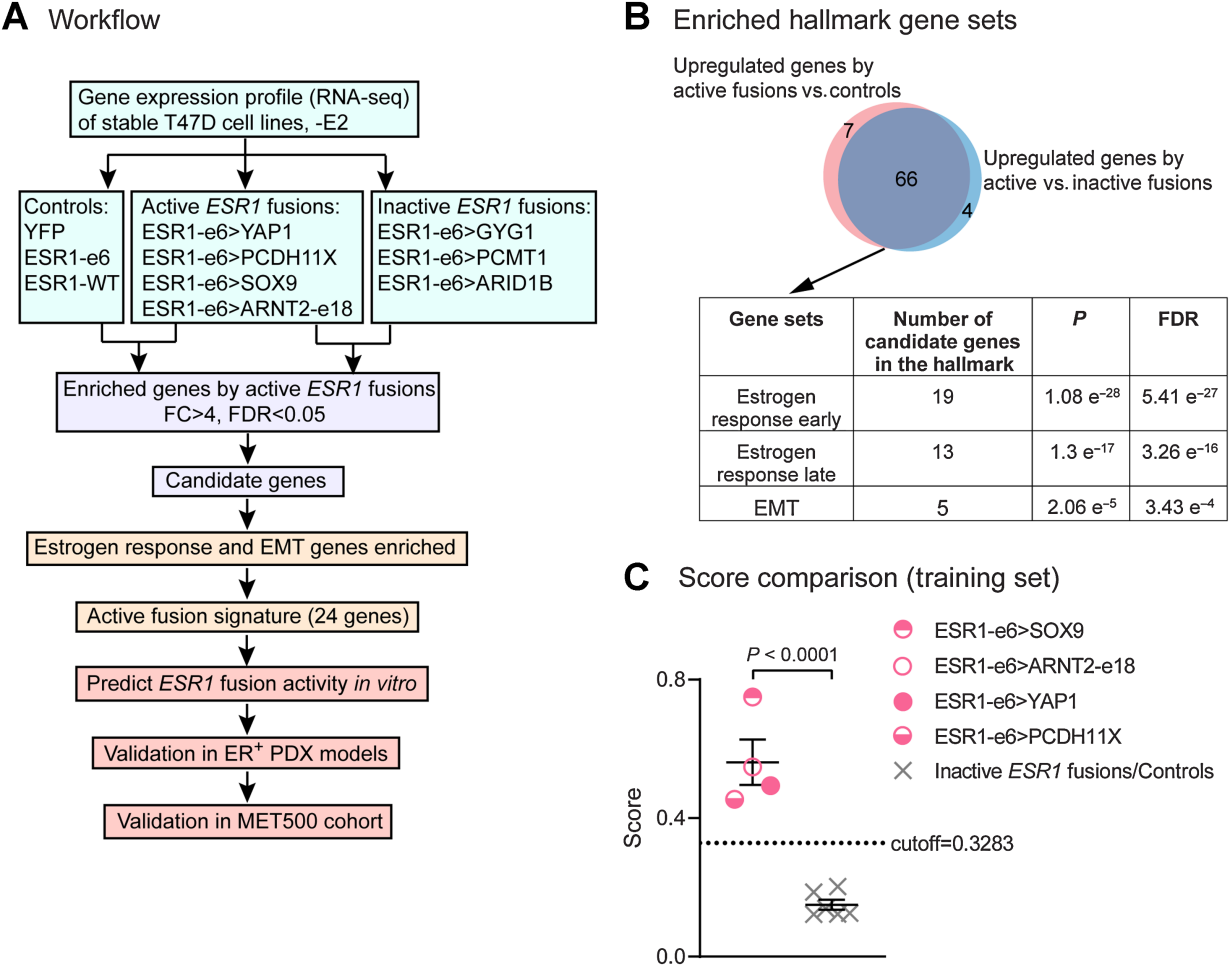
Active ESR1 fusions program a unique, 24-gene transcriptional signature. **A,** Workflow to identify the gene signature to predict active fusions. FC, fold change; FDR, false discovery rate. **B,** Venn diagram showing overlapping upregulated genes by active ESR1 fusions compared with inactive fusions or control cells. The table below shows the top three Hallmark gene sets enriched in the candidate genes. **C,** Scatter plot showing signature scores of active ESR1 fusions (ESR1-e6>YAP1, ESR1-e6>PCDH11X, ESR1-e6>SOX9, and ESR1-e6>ARNT2-e18) compared with inactive fusions (ESR1-e6>GYG1, ESR1-e6>PCMT1, and ESR1-e6>ARID1B) and control cells (YFP, ESR1-e6, and ESR1-WT) all minus E2. A two-tailed *t* test was used to calculate statistical significance.

The 3′ partners of *ESR1* fusion genes are highly diverse; consequently, their presence is only revealed by unbiased genomic techniques such as whole-genome sequencing or RNA-seq. These techniques are not routinely used clinically, and it is currently unknown how sensitive unbiased techniques are as screens for an *ESR1* gene fusion event, because an orthogonal assay is requited to determine sensitivity. Adding to diagnostic complexity, some ESR1 fusion proteins are inactive and therefore not clinically actionable. An *in vitro* assay such as the ones described above are feasible but difficult to conduct within a clinically useful time-frame. We therefore sought to develop a gene expression signature that is diagnostic for the presence of a transcriptionally active ESR1 fusion protein. RNA-seq was applied to T47D cells expressing *ESR1* fusion cDNAs to identify genes that were selectively upregulated by the four transcriptionally active ESR1 fusion proteins as compared with: (i) three inactive ESR1 fusions and (ii) three controls (Fig. 4A). These two comparisons yielded an overlapping group of 66 candidate genes with a fold change greater than 4 and a false discovery rate (FDR) less than 0.05. Overrepresentation analysis using Hallmark pathways from MSigDB (25, 26) identified candidate genes that were overrepresented in the estrogen response (early and late) and EMT gene sets (Fig. 4B). An active ESR1 fusion signature was then devised based on estrogen response and EMT genes, as these were the top two pathways modulated by expression of an active ESR1 fusion protein. Specifically, we identified 24 Hallmark genes, including 19 genes in the estrogen response set (*CHST8*, *MAPT*, *OLFM1*, *PDZK1*, *RASGRP1*, *MPPED2*, *GREB1*, *MYB*, *GFRA1*, *PGR*, *ELOVL2*, *ADCY1*, *NPY1R*, *TFF1*, *ACOX2*, *SGK1*, *STC2*, *CALCR*, and *KRT13*), two genes in the EMT gene set (*VCAN* and *COL3A1*), and three genes in both gene sets (*CXCL12*, *GJA1*, and *TGM2*). The expression of each gene was ranked by percentile within each sample and scores were computed as the mean percentile of the signature gene sets. *ESR1* fusions were predicted as encoding active or inactive proteins according to the cutoff obtained by the ROC curve analysis (cutoff, 0.3283; Supplementary Fig. S4). In this training set, transcriptionally active ESR1 fusion proteins showed significantly higher scores as compared with inactive fusions and controls, as expected (Fig. 4C).

### The 24-gene transcriptional signature predicts the *in vitro* activity of additional *ESR1-e6* fusion genes

**Figure 5.**
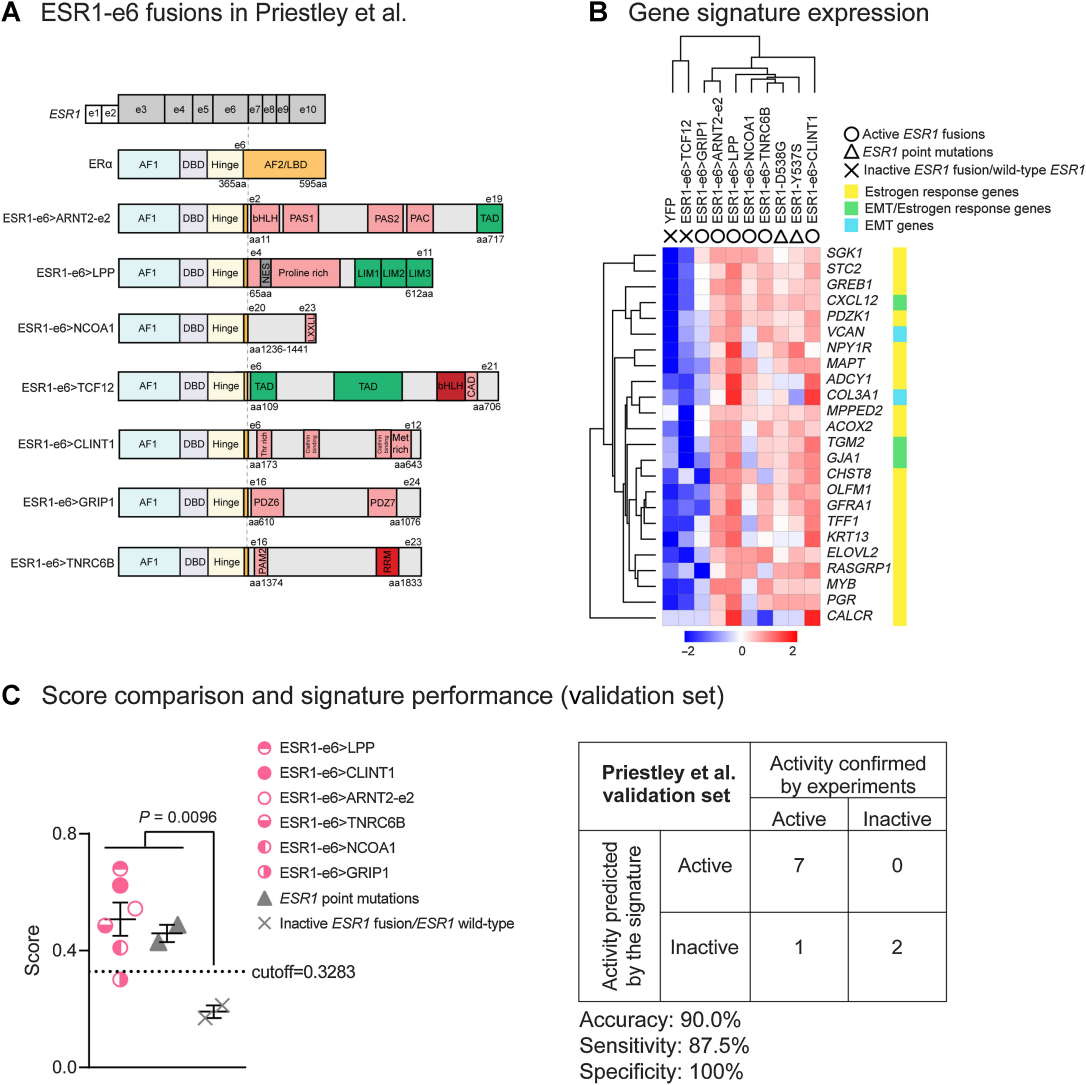
The MOTERA signature predicts activity of additional *ESR1* fusions identified in ER^+^ MBC patients. **A,** Seven additional ESR1-e6 fusions identified in Priestley et al. (27) are illustrated. These in-frame fusions possess a common structure as shown in Fig. 1B. Pink boxes represent protein–protein interactions, including the Per-Arnt-Sim (PAS) domain, PAC motif, LXXLL motif, class A specific domain (CAD), threonine-rich domain (Thr rich), methionine-rich domain (Met rich), PDZ domain, and PABPC1-interacting motif-2 (PAM2). Green boxes either represent transcriptional activation domains (TAD) or LIM zinc-binding (LIM) domains that provide coactivator function for LPP. The gray box represents a nuclear export signal (NES) in LPP. Red boxes represent the bHLH DNA binding domain and the RNA recognition motif (RRM). **B,** Heatmap showing the expression of the 24-gene signature in T47D cells expressing additional ESR1 fusions and LBD point mutations (Y537S and D538G). Scale bar indicates row Z scores. **C,** Left, scatter plot showing signature scores of *ESR1* mutations (including fusions and LBD point mutations) and YFP control cells expressing endogenous ERα. Two-tailed *t* test was used to compare scores. The ESR1–GRIP1 fusion was the only active fusion that did not reach score significance. Right, confusion matrix to measure the performance of the signature to predict the activities of ESR1 fusions. Accuracy is the proportion of correctly predicted events in all cases. Sensitivity is the ability of the signature to predict an active fusion event to be active. Specificity is the ability of the signature to predict an inactive fusion event to be inactive.

To validate the 24-gene ESR1 fusion activity signature, we studied seven additional *ESR1* gene fusions published by Priestly and colleagues (27). These in-frame ESR1-e6 fusions were identified in ER^+^ MBC patients by whole-genome sequencing, including four fusions with TF or CoA partners, ESR1-e6>ARNT2-e2, ESR1-e6>LPP, ESR1-e6>NCOA1, and ESR1-e6>TCF12 (Fig. 5A). Another three fusions, analogous to *PCDH11X*, involved genes encoding protein–protein interaction motifs that serve nontranscriptional cellular functions, including ESR1-e6>CLINT1, ESR1-e6>GRIP1, and ESR1-e6>TNRC6B. The same approach as in Fig. 2 was taken to assess the function of these new fusions *in vitro*. All of the seven *ESR1* fusion cDNAs expressed stable chimeric proteins in T47D and MCF7 cells (Supplementary Fig. S5A and S5B). Three fusions that involve TF/CoA partners, ESR1-e6>ARNT2-e2, ESR1-e6>LPP, and ESR1-e6>NCOA1, drove E2-independent and fulvestrant-resistant growth, as well as increased motility of T47D cells, when compared with the YFP controls (–E2, +DMSO; Supplementary Fig. S5C–S5E). Surprisingly, the fourth fusion, ESR1-e6>TCF12, which involves a TF in the basic helix–loop–helix (bHLH) E-box family, expressed a stable chimeric protein, but was inactive in both T47D and MCF7 cells (Supplementary Fig. S5C–S5E). The ESR1-e6>TCF12 fusion was able to bind to concatenated EREs in a pulldown assay similar to active fusion examples (ESR1-e6>YAP1, ESR1-e6>SOX9, and ESR1-e6>CLINT1; Supplementary Fig. S6), thus suggesting that the transcriptional inactivity of ESR1-e6>TCF12 was not due to lack of an ability to bind DNA.

Three gene fusions that did not involve a known TF/CoA partner, ESR1-e6>CLINT1, ESR1-e6>GRIP1, and ESR1-e6>TNRC6B, but all demonstrated ET-resistant cell growth and enhanced E2-independent motility, although the effect of ESR1-e6>GRIP1 on proliferation was statistically marginal (Supplementary Fig. S5C–S5E). RNA-seq was then performed on RNA extracted from T47D cells that expressed each new ESR1 fusion protein, as well as on RNA from YFP control cells. In this experiment, we also included two common *ESR1* LBD point mutations (Y537S and D538G) to compare the active ESR1 fusion signature with the transcriptional profile associated with known activating ESR1 point mutants. Five out of six active ESR1 fusions (ESR1-e6>ARNT2-e2, ESR1-e6>LPP, ESR1-e6>NCOA1, ESR1-e6>CLINT1, and ESR1-e6>TNRC6B) demonstrated similar elevated expression of the 24-gene signature in sum, although there was some variability at the level of individual genes (Fig. 5B). ESR1-e6>GRIP1 induced lower expression of the 24-gene signature than other active fusion examples, consistent with its weaker activity in proliferation assays compared with the five other fusions studied. Interestingly, the two ESR1 LBD point mutant proteins expressed in T47D cells induced similar levels of gene expression from the 24-gene signature as active ESR1 fusion proteins, suggesting that despite different mutational mechanisms for ESR1 protein activation, LBD point mutants and translocated ERs activate a similar pathogenic transcriptional pattern (Fig. 5B). The mean signature scores of active ESR1 fusions and LBD point mutants were significantly increased compared with those of the inactive ESR1-e6>TCF12 fusion and YFP (endogenous ERα) control (Fig. 5C). As expected, the mean score of the weakly active ESR1-e6>GRIP1 fusion fell below the cutoff value. The validation statistics of the independent Priestley and colleagues (27) set showed an accuracy of 90.0% (specificity, 100%; sensitivity, 87.5%; Fig. 5C). Because the 24-gene signature was similarly induced by ESR1 LBD point mutants and active ESR1 fusion proteins, it was given the moniker “MOTERA” for Mutant or Translocated Estrogen Receptor Alpha.

### The MOTERA signature accurately predicts the presence and functional status of *ESR1* mutations and gene fusions in ER^+^ PDX tumors and clinical samples.

**Figure 6.**
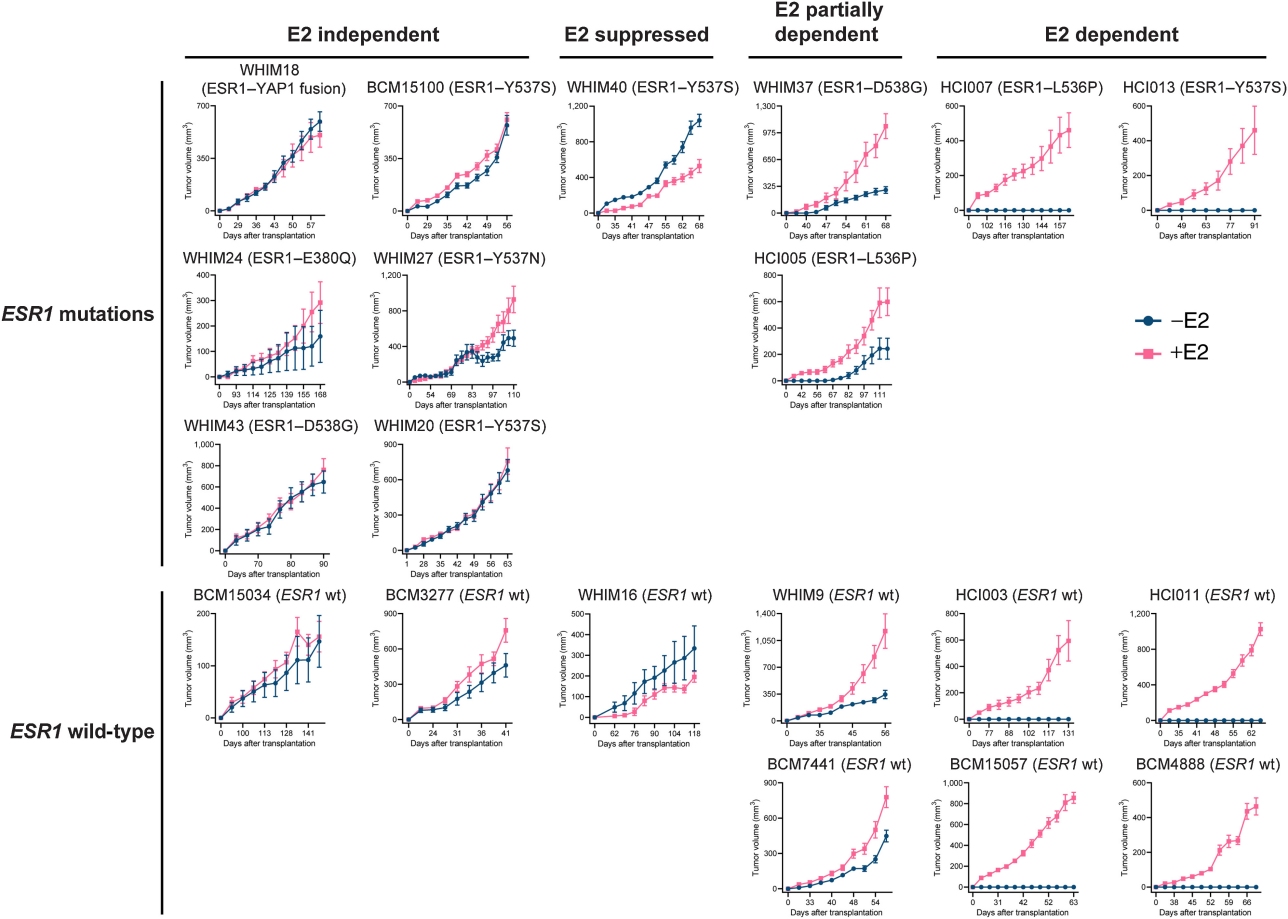
The growth of 20 ER^+^ PDX tumors in xenografted mice in the absence and presence of E2. Volumes of 20 ER^+^ PDX tumors were measured in ovariectomized SCID/beige mice supplemented with or without 8 μg/mL E2 in the drinking water (mean ± SEM; *n* = 7–16 per PDX line per arm). PDX tumors were categorized based on *ESR1* status (mutations listed or wild-type, wt) and E2 dependency for growth (E2 independent, E2 suppressed, E2 partially dependent, and E2 dependent).

**Figure 7.**
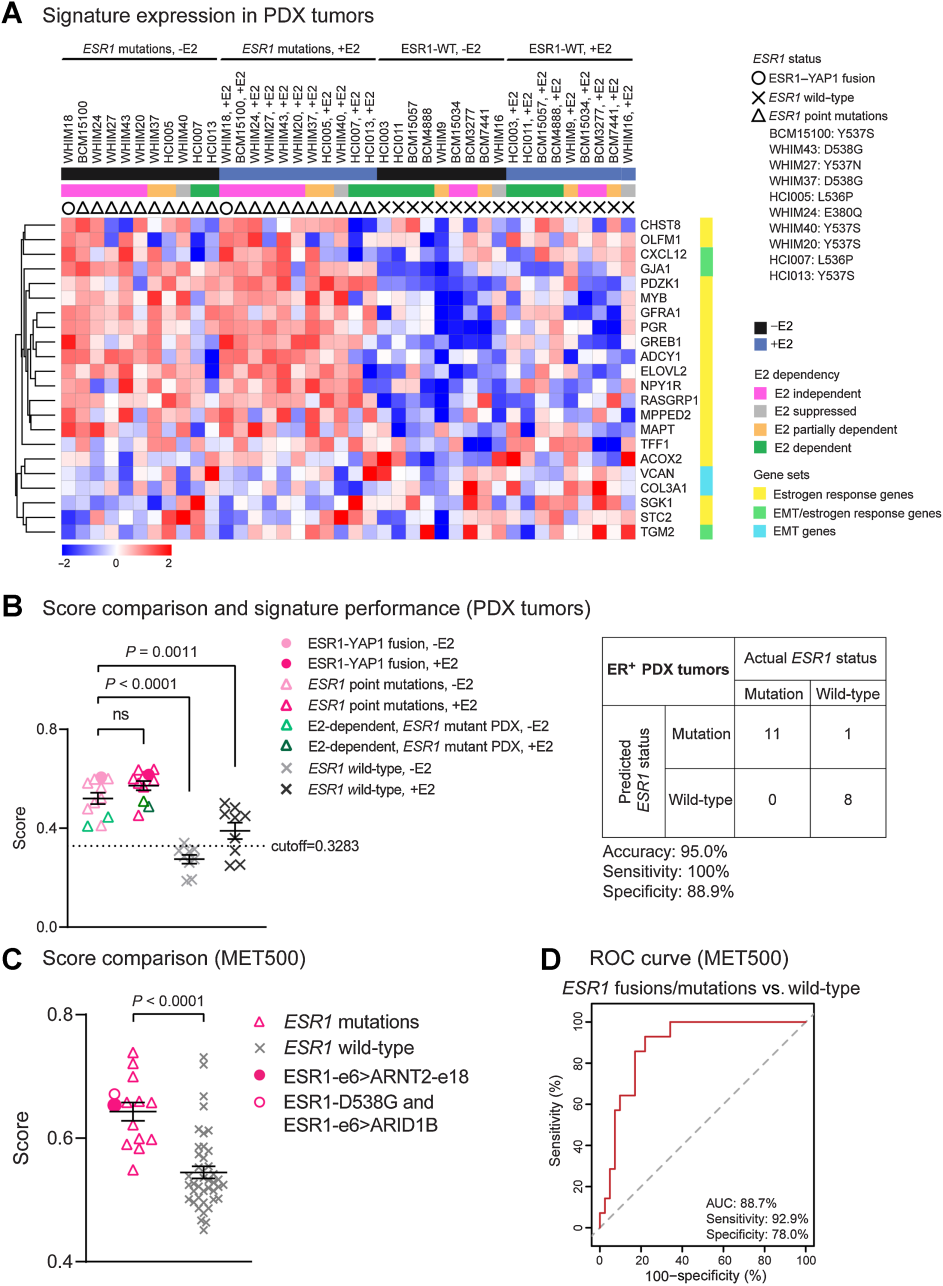
The MOTERA signature predicts activity of *ESR1* fusions/point mutations in ER^+^ PDX tumors and in MBC patients. **A,** Heatmap showing the expression of the 24-gene signature in 20 ER^+^ PDX tumors. Scale bar indicates row *Z* scores. *CALCR* and *KRT13* in the signature were missing in the PDX RNA-seq data, so they were not included in the heatmap. **B,** Left, scatter plot showing mean signature scores of *ESR1* mutations (including the ESR1–YAP1 fusion and LBD point mutations) and ESR1-WT expressing tumors. One-way ANOVA with Dunnett multiple comparisons test was used to calculate statistical significance. Right, confusion matrix to measure the performance of the signature to predict the presence of *ESR1* mutations. Accuracy is the proportion of correctly predicted events in all cases. Sensitivity is the ability of the signature to predict an *ESR1* mutation to be a mutant. Specificity is the ability of the signature to predict an ESR1-WT to be wild-type. **C,** Scatter plot showing mean signature scores of MBC patient tumors expressing *ESR1* mutations versus ESR1-WT in the MET500 cohort (9). Two-tailed *t* test was used to compare scores. **D,** ROC curve for the 24-gene signature performance to differentiate *ESR1* mutations from ESR1-WT in the MET500 cohort. The AUC is the probability that the signature ranks a randomly chosen *ESR1* mutation higher than a randomly chosen WT *ESR1* (100% is the best test, and the dashed diagonal line illustrates the performance of a random signature).

To test the properties of the MOTERA signature in human tumors that naturally express either *ESR1* gene fusions or mutations, we examined performance for *ESR1* fusion or point mutation detection in a panel of 20 ER^+^ PDX tumors. The E2 dependence of each PDX tumor was evaluated in ovariectomized SCID/beige mice with or without 8 μg/mL E2 in the drinking water (Fig. 6). *ESR1* mutation status was determined by WES and gene expression was determined by RNA-seq under both plus estradiol (+E2) and minus estradiol (–E2) conditions. When tumors were completely E2 dependent, the –E2 transcriptome was established by replacing the +E2 water with control (–E2) water for one week and then harvesting the tumors. As expected, the MOTERA signature was highly expressed in the E2-independent WHIM18 PDX naturally expressing the ESR1–YAP1 fusion protein (6) (Fig. 7A), thus demonstrating a high degree of similarity between the experimental context of the *ESR1-e6>YAP1* cDNA in T47D cells and the natural context in a PDX where this fusion was first identified. Consistent with T47D-based gene expression findings displayed in Fig. 5B, ET-resistant PDXs bearing a variety of *ESR1* LBD point mutations also induced the MOTERA signature, confirming an overlap between the transcriptional properties of active ESR1 fusion proteins and LBD point mutants noted in T47D cell experiments (Fig. 7A). For example, the MOTERA signature score was enriched over the cutoff derived from the T47D training set in the cases of BCM15100, WHIM20, WHIM40, and HCI013 (all expressing ESR1–Y537S), WHIM37 and WHIM43 (expressing ESR1–D538G), WHIM24 (expressing ESR1–E380Q), WHIM27 (expressing ESR1–Y537N), and HCI005 and HCI007 (expressing ESR1–L536P) mutants (Fig. 7B). PDX tumors expressing ESR1-WT (HCI003, HCI011, BCM15057, BCM4888, BCM15034, BCM3277, BCM7441, WHIM9, and WHIM16) had MOTERA scores below the cutoff in low estradiol (–E2) conditions in each case (Fig. 7B). We note that the mean signature scores for ESR1-WT tumors increased with E2, consistent with some genes in the signature being E2-induced (Fig. 7A and B). Thus, as a screening tool, the MOTERA signature is likely to be more specific if the biopsy sample is taken while the patient is taking an AI or an antiestrogen. Paradoxically, the HCI013 PDX example harbors the Y537S *ESR1* mutation but remained E2 dependent as previously reported by Welm and colleagues (Fig. 6; ref. 28). Similarly, HCI007 harbors an *ESR1 L536P* mutation, but also grew in an E2-dependent manner. These tumors have lower MOTERA scores but still above the training set defined cutoff. Presumably in these examples, ESR1-WT is functionally dominant over the LBD-mutant ERα, although the mechanism remains obscure. Under –E2 conditions, the MOTERA signature successfully distinguished between ET-resistant tumors driven by mutant or translocated ESR1 proteins from ESR1-WT PDXs, with an accuracy of 95.0% (specificity, 88.9%; sensitivity, 100%; Fig. 7B). Although the MOTERA transcriptional signature was largely composed of estrogen response genes, expression levels were not affected by E2 supplementation to the WHIM18 ESR1-YAP1 expressing PDX or other PDXs expressing *ESR1* LBD point mutations, underscoring sensitivity for the activated ESR1-mutant/translocated protein state (Fig. 7A and B). Upon E2 treatment, the MOTERA scores of ESR1-WT bearing PDX lines still remained significantly lower than those of *ESR1* mutated tumors, although in several cases, expression levels rose above the cutoff established in E2-deprived conditions (Fig. 7B).

An independent RNA-seq data set of 55 ER^+^ mRNA-positive MBC cases from the MET500 study (9) was used to further evaluate the performance of the MOTERA gene signature in tumor samples. Signature scores were significantly elevated in tumors expressing *ESR1* LBD point mutations, such as Y537S and D538G, versus ESR1-WT samples (Fig. 7C). Two *ESR1* fusions that were functionally studied in Fig. 2 (ESR1-e6>ARNT2-e18 and ESR1-e6>ARID1B) were both originally identified from the MET500 study. As expected, the ESR1-e6>ARNT2-e18 fusion drove a high MOTERA score in the sample in which it was identified. Against predictions, the functionally inactive ESR1-e6>ARID1B fusion also had a positive MOTERA signature score. However, this patient sample also harbored an *ESR1-D538G* LBD mutation, likely explaining the discordance. In terms of performance, the MOTERA signature score significantly distinguished active *ESR1* mutations (Y537S, D538G, and Y537C point mutations and the *ESR1-e6>ARNT2-e18* fusion) from WT *ESR1*, with a sensitivity of 92.9% and a specificity of 78.0% for an AUC of 88.7% (95% confidence interval, 80.0%–97.3%; Fig. 7D).

## Discussion

The data presented herein clearly demonstrate that most in-frame ESR1-e6 fusion proteins derived from interchromosomal translocations are drivers of ET resistance. Hitherto the clinical importance of *ESR1* gene translocation has been underappreciated because the diversity of C-terminal partner genes creates a considerable diagnostic challenge. Fluorescence *in situ* hybridization (FISH) and PCR approaches that require the identification of both partners in a gene fusion event are not applicable. Even in the case of ESR1-e6>ARNT2, where we identified two examples, the *ARNT2* exons present at the fusion junctions were different. Break-apart FISH probes can be considered in a setting where only one partner in the fusion is known. However, this approach does not identify the unknown 3′ partner gene or the reading frame, which is critical because inactive out-of-frame fusions are common (Supplementary Table S1). RNA-seq is clearly an applicable unbiased discovery approach, but sensitive detection requires the identification of a sufficient number of fusion junction reads to confidently diagnose the presence of an in-frame translocation. When RNA-seq coverage is low, or the RNA is of low quality, fusion junction sequences could easily remain undetected.

Adding to the difficulty of understanding the clinical significance of *ESR1* gene fusions is the fact that only a subset of ESR1 fusion proteins are active, and therefore clinically actionable. Consistent rules to diagnose whether a fusion is active based on the known functions of the C-terminal fusion partners proved hard to define. Although *ESR1-e6* fusions with *YAP1*, *SOX9*, *ARNT2*, *LPP*, and *NCOA1* are all known positive regulators of transcription and produce active fusion proteins, our analysis of the ESR1-e6>TCF12 fusion protein produced an interesting exception. *TCF12* encodes a bHLH E-box TF, and its two TADs (29) are present in the fusion. Nonetheless, the synthetic *ESR1-e6>TCF12* cDNA was inactive in both T47D and MCF7 cells. We cannot exclude the possibility that this particular fusion is only active in the context of the cancer in which it evolved, i.e., the indicator cell lines we used lack the requisite coactivators. If truly inactive, however, the *ESR1-e6>TCF12* fusion event raises the question of how this example could have been selected during clonal evolution. A potential explanation is provided by the ESR1-e6>ARID1B fusion protein, which is transcriptionally inactive with a 3′ partner gene related to the established tumor suppressor *ARID1A* (30, 31). It has been proposed that *TCF12* encodes a tumor suppressor (29, 32). Thus, selection of transcriptionally inactive *ESR1* fusions could be explained if these fusions inactivate tumor suppressor functions encoded by the 3′ partner gene. One could even speculate that these putative ESR1 tumor suppressor fusion proteins act in a dominant-negative fashion, thereby interrupting the function of the remaining intact TCF12 or the ARID1A activity. Multiple active non-TF/CoA fusions (*PCDH11X*, *DAB2*, *CLINT1*, *GRIP1*, and *TNRC6B*) dramatically add to the complex landscape of *ESR1* fusion genes. The activity of these fusions cannot, by definition, be predicted from an understanding of the normal function of each 3′ partner gene involved because none are known to be a TF or CoA, and the WT protein is not nuclear localized. Presumably the fusion partners have diverse protein–protein interaction domains that are subverted for the purposes of activating gene transcription in the context of a pathologic fusion with *ESR1*. These questions must be addressed in follow-up mechanistic studies.

One diagnostic approach after the detection of an in-frame *ESR1* fusion gene would be to test the newly identified example *in vitro*. However, this is inefficient for clinical care and may not always produce an accurate result. These concerns stimulated the development of the MOTERA gene signature to screen for tumors driven by the diverse somatic events that activate *ESR1* through the presence of a diagnostic gene signature. In a setting where an *ESR1*-activating mutation has already been identified, the MOTERA signature could be used to confirm whether the mutant *ESR1* gene is indeed driving ET resistance. However, the MOTERA signature is likely to be of most value in the setting where a canonical *ESR1* LBD point mutation has not been detected. Here, a high MOTERA score would warrant further investigation to detect a functional in-frame *ESR1-e6* fusion. Reflex diagnostic approaches for these cases could include unbiased RNA-seq, *ESR1*-specific 3′ rapid amplification of cDNA ends (3′-RACE) or break-apart *ESR1* FISH. Although break-apart FISH would not identify the C-terminal partner, its presence has already been signaled by a positive MOTERA score implying the unknown partner in the chimera is transcriptionally active.

Analysis of the MET500 data indicates that MBC with high MOTERA scores but without an *ESR1* point mutation detected by genome sequencing or translocation detected by RNA-seq are not infrequent (Fig. 7C). Possibilities for these cases include: (i) the RNA-seq result was false-negative for the presence of an active *ESR1* fusion; (ii) the exome sequencing was a false-negative for the presence of an *ESR1* mutation; (iii) the MOTERA score was a false-positive that reflects WT ERα activity because the sample was taken when the patient was not taking ET and the tumor was still E2 dependent; and (iv) some WT ERα MBC persist by expressing a similar MOTERA signature that might be driven by other mechanisms, like TFs other than ERα. The prospective evaluation of the MOTERA signature is therefore the next phase of our investigation.

An important focus for future studies will be to determine the clinical characteristics of *ESR1* fusion–driven tumors. Of particular interest is an examination of the metastatic spread associated with tumors expressing *ESR1* gene fusions, as in mouse xenograft systems, active ESR1 fusions drive lung metastasis (7). Distinct from WT ERα, active ESR1 fusions strongly induce EMT-related genes, which we functionally annotated using motility and invasion assays. This property differentiates the MOTERA signature from other gene sets that measure activity of the ERα pathway, such as the Hallmark early/late estrogen response gene set. Consistent with this, the MOTERA scores of *ESR1*-mutated PDX tumors were still significantly higher than those of *ESR1-WT*–bearing lines that received E2 treatment. Interestingly, EMT-related gene expression is elevated during mammary gland development as the nascent ducts invade the mammary fat pad, and then EMT gene expression is reduced after puberty (33, 34). Thus, active ESR1-e6 fusion proteins may be reactivating a developmental EMT program that is usually silenced in mature breast epithelial cells. Specific examples of ESR1 fusion–induced genes in the MOTERA signature that are related to metastasis include *SGK1*, which encodes serum- and glucocorticoid-inducible kinase 1 and promotes breast cancer bone metastasis (35). *VCAN* encodes versican, whose expression is significantly correlated with metastasis and poor overall survival (36). *GJA1* encodes connexin-43, a gap junction protein that mediates tumor cell migration and invasion (37, 38). *GFRA1* encodes GFRα that acts as a coreceptor in conjunction with the RET receptor, and activation of GFRα-RET signaling by binding the glial-derived neurotrophic factor (GDNF) ligand leads to ERα serine phosphorylation and enhanced transcriptional activity (39).

At least one *ESR1* fusion partner gene described herein has been observed in other settings. Gene fusions involving *LPP*, the gene encoding the Lipoma Preferred Partner protein, such as a recurrent *HMGA2–LPP* fusion have been found in multiple tumors, including lipoma (40), pulmonary chondroid hamartomas (41), and chondromas (42). In leukemia, an *MLL–LPP* fusion has been identified (43). Similar to the *ESR1-e6>LPP* fusion, these fusions preserve the three C-terminal LIM domains encoded by the *LPP* gene, which serve as the binding site for the ETS domain TF PEA3 and contain coactivator activity (44). It is therefore likely that in larger studies, some *ESR1* gene fusions will be observed to be recurrent, making the diagnosis of some *ESR1* translocations easier.

In conclusion, *ESR1-e6* gene fusions are part of the spectrum of the somatic mutations that constitutively activate ESR1 proteins in advanced ER^+^ breast cancer to drive poor outcomes. The MOTERA signature should be useful to answer the question how common these events are, because it will focus sensitive fusion detection approaches on cases where there is transcriptional evidence for an activating *ESR1* fusion (or mutation) that has not been diagnosed yet. Once the clinical significance of *ESR1* gene fusions becomes more widely recognized and the diagnostic approach becomes more efficient, specific treatment approaches for tumors expressing active ESR1 fusion proteins can be developed.

## Authors' Disclosures

X. Gou reports grants from Cancer Prevention and Research Institute of Texas (CPRIT) Training Grant during the conduct of the study; in addition, X. Gou has a patent for 24-gene diagnostic signature pending to BCM. L.E. Dobrolecki reports personal fees from StemMed, Ltd., outside the submitted work. M.T. Lewis reports grants from NCI and CPRIT during the conduct of the study and other support from StemMed Ltd and Tvardi Therapeutics Inc. outside the submitted work. A.L. Welm reports other support from Thunder Biotech, Modulus Therapeutics, Inc., and J. Michael Bishop Institute for Cancer Research outside the submitted work; and A.L. Welm may receive tangible property royalties if University of Utah chooses to license HCI PDX models to for-profit-companies. S. Li reports grants, personal fees, and nonfinancial support from NIH 5U54CA224083 during the conduct of the study and personal fees from Envigo outside the submitted work. C.E. Foulds reports grants from Adrienne Helis Malvin Medical Research Foundation during the conduct of the study; other support from Coactigon, Inc., outside the submitted work; in addition, C.E. Foulds has a patent for 24-gene diagnostic signature pending to BCM. M.J. Ellis reports grants from NCI, Susan G. Komen Foundation, DOD Breast Cancer Research Program, McNair Medical Institute at The Robert and Janice McNair Foundation, CPRIT, and Adrienne Helis Malvin Medical Research Foundation during the conduct of the study; personal fees from Lilly, Novartis, AstraZeneca, Pfizer, Veracyte, Bioclassifier, and Washington University in St Louis outside the submitted work; in addition, M.J. Ellis has a patent for MOTERA signature pending. No disclosures were reported by the other authors.

## Supplementary Material

Supplementary Data It includes Supplementary Methods, Supplementary Figures, Supplementary Tables, and Supplementary References
