# Supplementary material for: Transcriptional Reprogramming Differentiates Active from Inactive ESR1 Fusions in Endocrine Therapy-Refractory Metastatic Breast Cancer
Source: Cancer Res. 2021 Oct 28;81(24):6259–72. doi: 10.1158/0008-5472.CAN-21-1256 (PMC9234971; doi:10.1158/0008-5472.CAN-21-1256)
Supplement: Supplementary Data — It includes Supplementary Methods, Supplementary Figures, Supplementary Tables, and Supplementary References [file can-21-1256_supplementary_data_suppsmsf1-sf6st1sr.docx]

**Supplementary Information**

**Transcriptional reprogramming differentiates active from inactive ESR1 fusions in endocrine therapy-refractory metastatic breast cancer**

Xuxu Gou, Meenakshi Anurag, Jonathan T. Lei, Beom-Jun Kim, Purba Singh, Sinem Seker, Diana Fandino, Airi Han, Saif Rehman, Jianhong Hu, Viktoriya Korchina, Harshavardhan Doddapaneni, Lacey E. Dobrolecki, Nicholas Mitsiades, Michael T. Lewis, Alana L. Welm, Shunqiang Li, Adrian V. Lee, Dan R. Robinson, Charles E. Foulds, Matthew J. Ellis*

**List of Supplementary Material**

**1) Supplementary Methods**

**2) Supplementary Figures**

- Supplementary Figure 1, related to Figure 2
- Supplementary Figure 2, related to Figure 2
- Supplementary Figure 3, related to Figure 3
- Supplementary Figure 4, related to Figure 4
- Supplementary Figure 5, related to Figure 5
- Supplementary Figure 6, related to Figure 5

**3) Supplementary Tables**

- Supplementary Table 1: Comprehensive summary of *ESR1* gene fusions in ER+ breast cancer

**4) Supplemental References**

**1) Supplementary Methods**

***Cell Culture***

All cell lines were obtained from the Tissue Culture Core at BCM in 2017, which originally came from ATCC. Cells were cultured at 37°C in 5% CO2 and were examined for mycoplasma every 6 months. In all experiments, the cells were no longer used after passage 20. T47D (ATCC Cat# HTB-133, RRID: CVCL_0553) and MCF7 (ATCC Cat# HTB-22, RRID: CVCL_0031) cells were grown in RPMI1640 with L-Glutamine (Corning, cat#10-040-CV) supplemented with 10% FBS (Sigma-Aldrich, cat#F8067), glucose to 4.5 g/L (Sigma, cat# G5767), 10 mM HEPES (GenDEPOT, cat# CA011), 1 mM sodium pyruvate (GenDEPOT, cat# CA017), and 50 μg/mL gentamycin (GenDEPOT, cat# CR003-001). For hormone deprivation, cells were plated in culture media overnight, washed with Dulbecco’s PBS, and maintained in phenol red-free, RPMI media (Fisher Scientific, cat#11835) containing charcoal stripped serum (Sigma, cat#F6765) as supplemented as above (CSS media). CSS media was changed every 2-3 days for 1-2 weeks. HEK293T (ATCC Cat# CRL-3216, RRID: CVCL_0063) cells were grown in DMEM with high-glucose (4.5 g/L), L-glutamine and sodium pyruvate (Mediatech, cat#10-013-CV) supplemented with 10% FBS (Sigma, cat#F8067) and 1% penicillin-streptomycin (Sigma, cat#P4333).

***Subcloning of ESR1 fusions into a lentiviral expression vector***

HA-tagged cDNAs encoding ESR1-e6>DAB2, ESR1-e6>GYG1, ESR1-e6>SOX9, ESR1-e6>ARNT2-e18, ESR1-e6>PCMT1 and ESR1-e6>ARID1B were subcloned from a pcDNA 3.1(+) vector (Gene Universal) into pCDH-CMV-MCS-EF1a-Puro lentiviral vector (System Biosciences, cat#CD510B-1) by restriction digestion and ligation with T4 DNA ligase, followed by DNA sequencing for verification. The synthesis, subcloning, and sequence verification of additional *ESR1* fusion cDNA constructs (ESR1-e6>ARNT2-e2, ESR1-e6>LPP, ESR1-e6>NCOA1, ESR1-e6>TCF12, ESR1-e6>CLINT1, ESR1-e6>GRIP1 and ESR1-e6>TNRC6B) into pCDH-CMV-MCS- EF1a-Puro lentiviral vector was performed by Gene Universal.

***Generation of lentiviral stable ESR1 fusion expressing cell lines***

The culture media containing lentiviral particles were collected after 48 hours, filtered using 0.45 μm pore filter (Millipore), and added to T47D (MOI=1) and MCF7 (MOI=2) cells in the presence of 8 μg/ml polybrene (Millipore, cat#TR-1003-G).

***Immunoblotting and immunoprecipitation***

Cells were harvested by scraping in cold PBS. Cell pellets were resuspended in either RIPA lysis buffer (Thermo Fisher Scientific, cat#89900) or MIB lysis buffer (50 mM HEPES (pH 7.5), 150 mM NaCl, 1 mM EDTA, 1 mM EGTA with 0.5% Triton X-100) supplemented with 1x protease inhibitors and 1x phosphatase inhibitors, and lysed on ice for 30 min or sonicated for 2 minutes on a Corvaris sonicator, respectively. Cell lysates were clarified by centrifugation at 20,000x g for 30 minutes at 4°C. Protein concentrations were determined by Bradford assay (Bio-Rad). Proteins (20 μg) were mixed with LDS Sample Buffer (Invitrogen, cat#NP0007), separated on 4%–12% gradient gels (Thermo Fisher Scientific), transferred to nitrocellulose membrane. Primary antibodies used for immunoblotting were as follows: N-terminal ERα (Millipore, Cat#04-820; RRID: AB_1587018, 1:1000), C-terminal ERα (Cell Signaling Technology Cat#8644, RRID: AB_2617128, 1:1000), Snail (Cell Signaling Technology, Cat#3879; RRID: AB_2255011, 1:1000), E-Cadherin (Cell Signaling Technology, Cat#14472; RRID: AB_2728770, 1:1000), GAPDH (Santa Cruz Biotechnology Cat# sc-47724, RRID: AB_627678, 1:5000) and DNA-PK_CS_ (Santa Cruz Biotechnology Cat# sc-5282, RRID: AB_2172848, 1:1000). The following HRP-conjugated secondary antibodies (anti-rabbit IgG, Cell Signaling Technology Cat# 7074, RRID: AB_2099233, 1:10,000) and anti-mouse IgG (Cell Signaling Technology Cat# 7076, RRID:AB_330924, 1:10,000) were employed and membranes were developed using ECL Detection Reagent (Fisher Scientific, cat#RPN2235) with visualization on a Bio-Rad Imaging System.

Hormone-deprived stable T47D cells were treated with or without 100 nM E2 for 45 minutes at 37°C, harvested and lysed in IP lysis buffer (Thermo Fisher Scientific, cat#87787) containing 1x protease inhibitors and 1x phosphatase inhibitors. Whole cell lysates (2 mg) were immunoprecipitated with 2 µg anti-HA tag antibody (Santa Cruz Biotechnology, mouse, Cat#sc-7392; RRID: AB_627809), or mouse IgG Control (Cell Signaling Technology Cat# 61656, RRID: AB_2799613) overnight at 4°C with rotation, followed by incubation of protein A magnetic beads (Bio-Rad, cat# 1614013) for 1h at 4°C and washing with IP lysis buffer for 3 times.

***Cell growth and motility assays***

Hormone-deprived stable cells were plated in 96-well plates (3,000 cells/well). One day after plating, cells were treated with or without 100 nM fulvestrant in the presence or absence of 10 nM E2. CSS media containing drugs were changed every 2-3 days. Cell growth was quantified using an alamarBlue reagent (resazurin sodium salt, Sigma, R7017, 0.25 mg/ml) after 7-10 days of treatment, and the plates were read by taking the ratio of 540 nm (λ_Excitation_)/ 590 nm (λ_Emission_) with a fluorescent microplate reader (BMG LABTECH).

Cell motility was detected using a scratch wound assay. Hormone-deprived cells were plated at 70,000 cell/well in a 96-well ImageLock plate overnight, and then pre-treated with mitomycin C before being subjected to wounding by a WoundMaker (Essen BioScience). CSS media containing mitomycin C was changed every 24 hours.

***RNA-Seq and analysis***

The GARP core made mRNA libraries from T47D RNA samples using the Illumina TruSeq Stranded mRNA library preparation protocol (p/n 1000000040498 v00, Oct 2017) along with ThermoFisher’s ERCC RNA Spike-In Control Mixes Protocol (p/n 4455352, rev. D) using 250 ng of starting total RNA. The resulting libraries were quantitated using the NanoDrop spectrophotometer and fragment size assessed with the Agilent Bioanalyzer. A qPCR quantitation was performed on the libraries to determine the concentration of adapter ligated fragments using the Applied Biosystems ViiA7 Real-Time PCR System and a KAPA Library Quant Kit (p/n KK4824). All samples were pooled equimolarly and re-quantitated by qPCR, and also re-assessed on the Bioanalyzer. Using the concentration from the ViiA7 TM qPCR machine above, 150 pM of equimolarly pooled library was loaded onto one lane of the NovaSeq S1 v1.5 flowcell (Illumina p/n 20028318) following the XP v1.5 Workflow protocol (Illumina kit p/n 20043130) and amplified by exclusion amplification onto a nanowell-designed, patterned flowcell using the Illumina NovaSeq 6000 sequencing instrument. PhiX Control v3 adapter-ligated library (Illumina p/n FC-110-3001) was spiked-in at 1% by weight to ensure balanced diversity and to monitor clustering and sequencing performance. A paired-end 100 bp cycle run was used to sequence the flowcell on a NovaSeq 6000 Sequencing System. An average of 25.7 million (M) read pairs per sample was sequenced.

For RNA-Seq on isolated ER+ PDX tumors, RNA-Seq was performed at the Human Genome Sequencing Center at BCM as follows. Strand-specific, poly-A+ enriched RNA-seq libraries for sequencing on the Illumina platform were prepared as described ([1](#_ENREF_1)). Briefly, poly-A+ mRNA was extracted from 1 μg total RNA, followed by fragmentation and first strand cDNA synthesis. The resultant cDNA was end-repaired, A-tailed and ligated with Illumina dual barcode adapters. Libraries were sequenced in groups of 70 on NovaSeq 6000 instrument using the S4 reagent kit (300 cycles) to generate 2 x 150 bp paired-end reads. Between 50 and 85M total reads were generated. The average strand-specificity and rRNA rate was 98% and 1.5%, respectively. The transcripts for 17,000 to 27,087 genes were detected in these samples.

Unsupervised hierarchical clustering was performed using a metric of Euclidean distance and the average linkage method. Differential expressed genes were analyzed using log(2)-transformed, upper quartile-normalized counts per million values with “edgeR” package in R ([2](#_ENREF_2)) comparing active *ESR1* fusions to inactive fusions and controls.

***Whole exome sequencing (WES) and ESR1 gene variant analysis***

WES data was generated by the Human Genome Sequencing Center at BCM using the Illumina platform. For this, paired-end libraries were constructed as described ([3](#_ENREF_3)) with the following modifications. Samples were barcoded at the ligation step using Illumina unique dual barcodes adapters (cat# 20022370) and were amplified 6-8 cycles using the Library Amplification Ready-mix containing KAPA HiFi DNA Polymerase (Kapa Biosystems, Inc). For capture enrichment, libraries were pooled in equimolar ratios in groups of 10 and were hybridized in solution to the HGSC VCRome 2.1 design. To this design, exome coverage across >3,500 clinically relevant genes that previously were covered below 20X (~2.72 Mb) was supplemented. Enriched libraries were sequenced on the NovaSeq 6000 instrument using the S4 reagent kit (300 cycles) to generate 2 x 150 bp paired-end reads. On average, 11.2 Gb of unique sequence data was generated with 95.3% of the bases in the exome design covered to 20x read depth or greater.

Somatic *ESR1* gene variants were called Strelka2, Mutect2, and CARNAC (v 0.2.5b9). Variants reported by these tools were filtered using GATK VariantFiltration (v 3.8.0) with parameters window 35, cluster 3, FS > 30.0, and QD < 2.0. We kept single nucleotide variants (SNVs) called by any two callers among Strelka2, Mutect2, and CARNAC. We filtered somatic SNVs by a minimal variant allele frequency (VAF) of 0.02. Then we used annovar (v 04.16.2018) to annotate the remaining variants.

***Immunofluorescence***

Hormone-deprived stable T47D cells were plated onto poly-D-lysine coated coverslips (Fisher) and grown overnight. Cells were fixed with 4% formaldehyde for 20 min at RT followed by permeabilization with 0.2% Triton X-100 for 10 min at RT and then blocked with 10% normal goat serum for 1h. HA-tagged ESR1 fusion proteins were detected with an anti-HA antibody (Cell Signaling Technology Cat# 2367, RRID:AB_10691311, 1:50) overnight at 4°C and then goat anti-mouse IgG secondary antibody (Alexa Fluor 568, Molecular Probes Cat# A-11004, RRID:AB_2534072, 1:1000) for 30 min at RT. Coverslips were mounted onto slides with ProLong Gold Antifade Reagent (Invitrogen). Fluorescence images were taken using a Nikon Eclipse Ti microscope equipped with a CoolSNAP EZ camera (Photometrics Scientific), a Plan Apo 40X/0.95 aperture objective and Nikon NIS elements software.

**2) Supplementary Figures**

**Supplementary Figure 1, related to Figure 2**

**
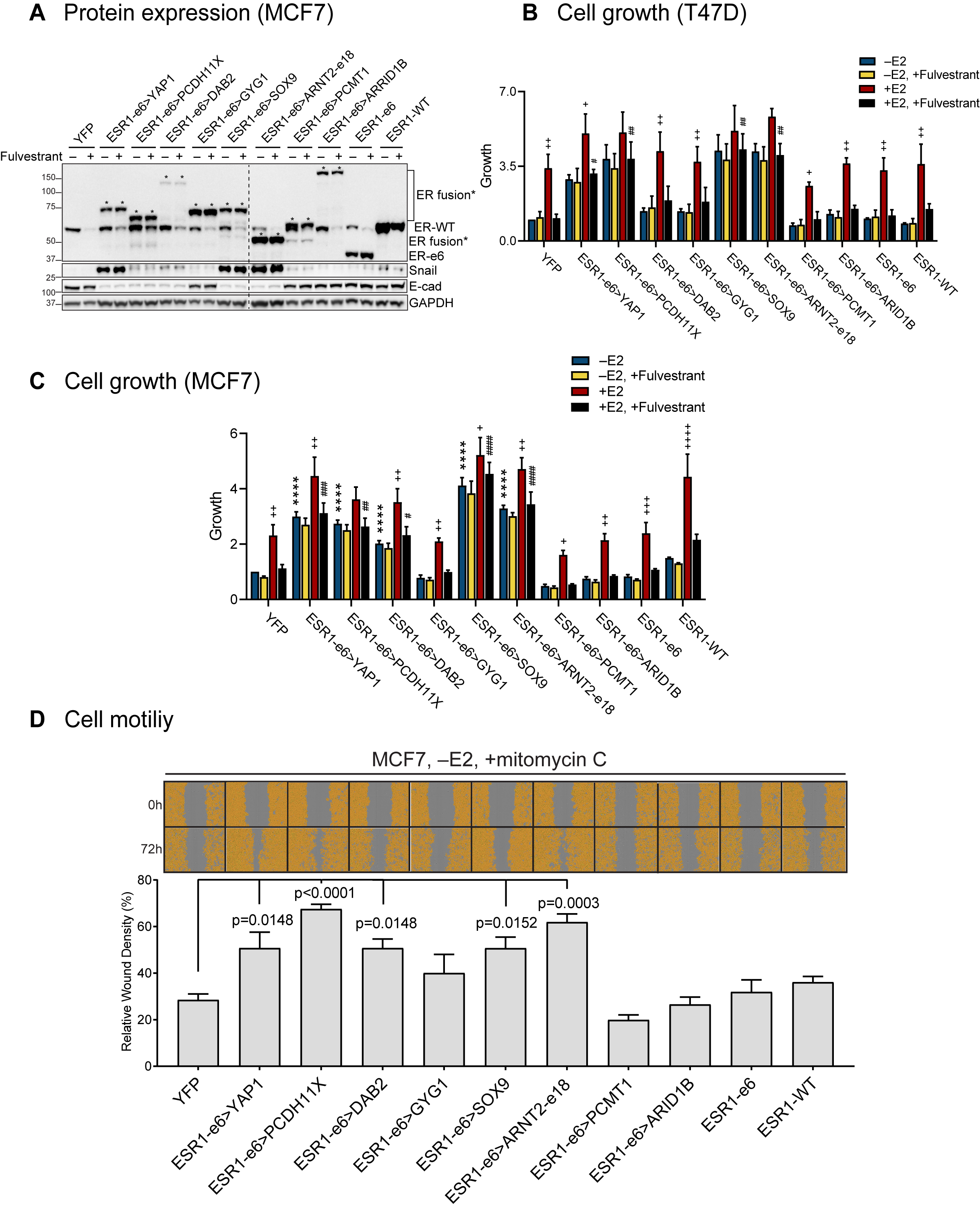
**

**Supplementary figure 1. In-frame ESR1 fusions drive ET-resistant cell growth and promote hormone-independent cell motility.** **(A)** Immunoblotting of ERα and ESR1 fusion proteins with an N-terminal ERα antibody, Snail or E-cad in hormone-deprived stably expressing MCF7 cells. GAPDH serves as a loading control. Asterisks indicate ESR1 fusion proteins. The dashed line indicates two separate blots that were conducted at the same time. The representative image is from two independent experiments. **(B)** Cell growth was assayed in stably expressing T47D cell controls (YFP, ESR1-e6, or ESR1-WT) or eight ESR1 fusion proteins (mean ± SEM, n=3). Two-way ANOVA test was used to compare each stable cell after 10 nM estradiol treatment (–E2 *vs* +E2, ^+^p<0.05, ^++^p<0.01, ^+++^p<0.001, and ^++++^p<0.0001). One-way ANOVA followed by Dunnett’s multiple comparisons test was used to compare each ESR1 fusion expressing cell line to YFP control cells in the presence of E2 and fulvestrant (+E2, +Fulvestrant) (^#^p<0.05, ^##^p<0.01, ^###^p<0.001 and ^####^p<0.0001). **(C)** Cell growth was assayed in MCF7 cells stably expressing various *ESR1* constructs (mean ± SEM, n=3). One-way ANOVA followed by Dunnett’s multiple comparisons test was used to compare each stable MCF7 cell line to YFP control cells. In the absence of estradiol (–E2), ****p<0.0001. In the presence of E2 and fulvestrant (+E2, +Fulvestrant), ^#^p<0.05, ^##^p<0.01, ^###^p<0.001 and ^####^p<0.0001. Two-way ANOVA was used to compare each stable cell line after 10 nM estradiol treatment (–E2 *vs* +E2, ^+^p<0.05, ^++^p<0.01, ^+++^p<0.001, and ^++++^p<0.0001). **(D)** Cell motility was detected using scratch wound assays in hormone-deprived stably expressing MCF7 cells, treated with 200 ng/ml mitomycin to block proliferation (mean ± SEM, n=3). One-way ANOVA followed by Dunnett’s multiple comparisons test was used to compare each cell line to YFP control cells. Cells are pseudo-colored orange to aid visualization.

**Supplementary Figure 2, related to Figure 2**

**Cell invasion**

**
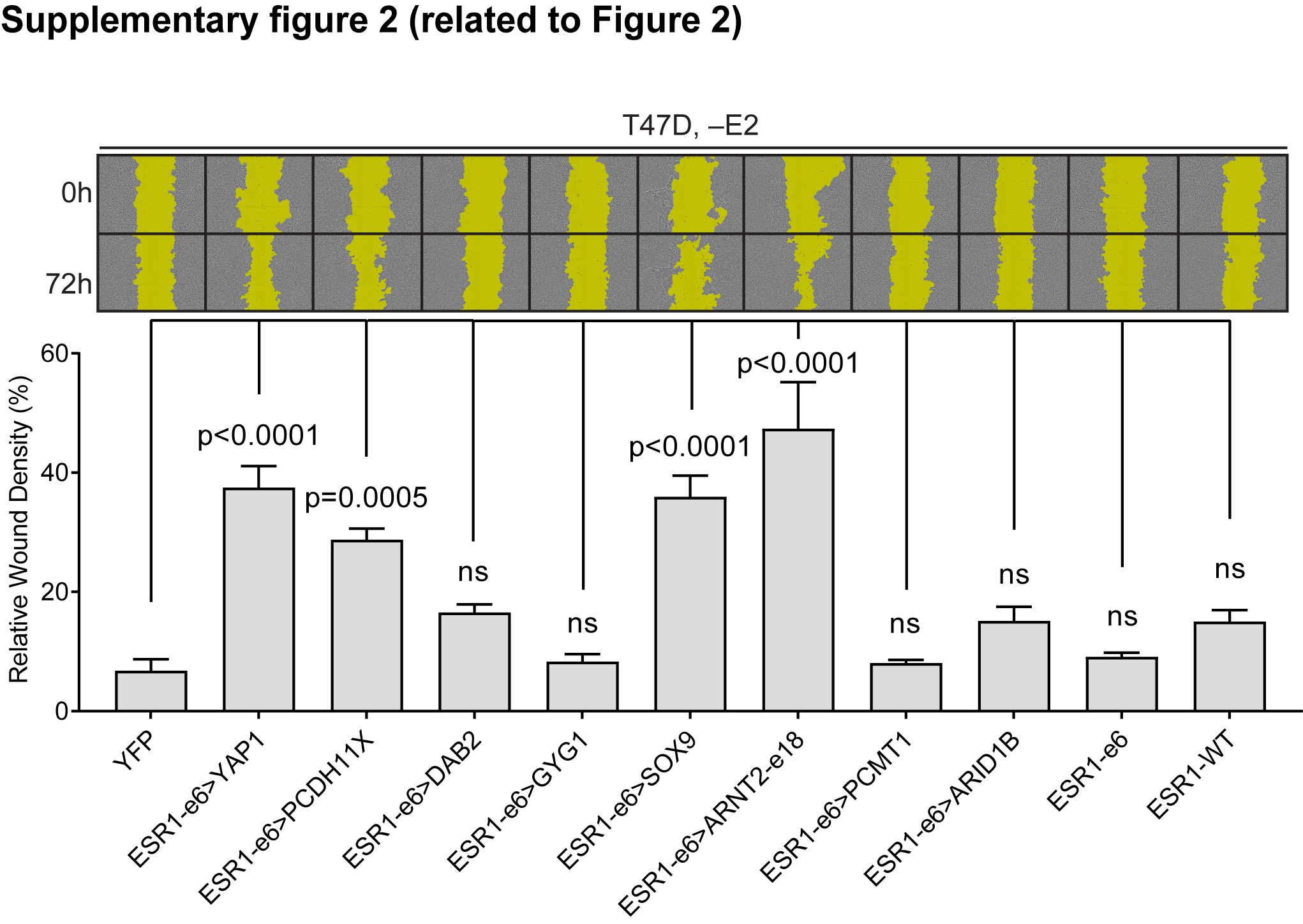
**

**Supplementary figure 2. Active in-frame ESR1 fusions promote hormone-independent cell invasion.** Cell invasion was detected in hormone-deprived stably expressing T47D cells, in a similar manner to the scratch wound assay except that cells were first plated on a Matrigel-coated plate, as described in the Methods. *Top panel*, IncuCyte images were recorded. *Bottom panel*, relative wound densities (%) were calculated and plotted (mean ± SEM, n=3). One-way ANOVA followed by Dunnett’s multiple comparisons test was used to compare each cell line to YFP control cells. Wound regions are pseudo-colored green to aid visualization.

**Supplementary Figure 3, related to Figure 3**


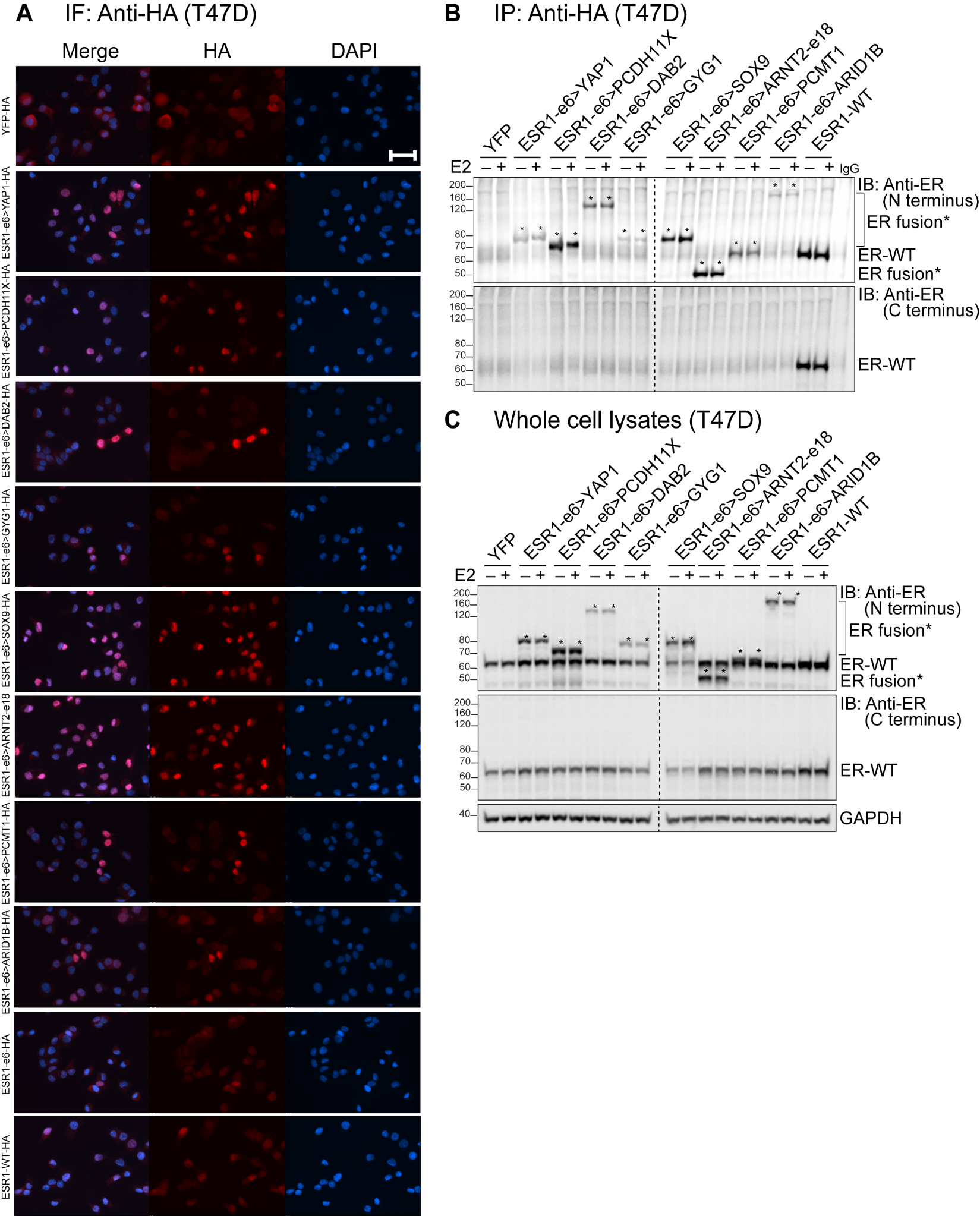


**Supplementary figure 3. ESR1 fusion proteins localize to the nucleus and do not form heterodimers with endogenously expressed ERα. (A)** Immunofluorescence staining with anti-HA tag antibody (red) on hormone-deprived stably expressing T47D cells showed nuclear localization of all eight HA-tagged *ESR1* fusion constructs. The nucleus was labeled with DAPI (blue). Representative images from two independent experiments are shown. Magnification 40X; Scale bar, 50 μm. **(B)** Hormone-deprived T47D cells stably expressing different *ESR1* constructs were treated with or without 100 nM E2 for 45 min. Cell lysates were then immunoprecipitated with an anti-HA antibody or mouse IgG control and then blotted with a N-terminal ERα antibody. Blotting with a C-terminal ERα antibody only detected ER-WT, and no co-immunoprecipitation with any ESR1 fusion protein. Representative images are from two independent experiments. The dashed line indicates two separate blots that were conducted at the same time. **(C)** Whole cell lysates (1% inputs for co-IP) were detected by immunoblotting with N- and C-terminal ERα antibodies, along with GAPDH as the loading control.

**Supplementary Figure 4, related to Figure 4**


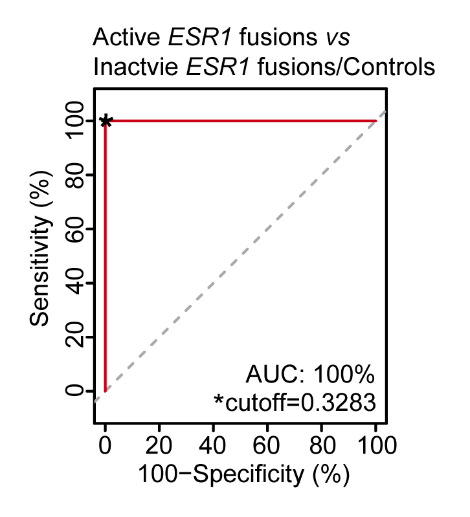


**Supplementary figure 4.** ROC curve for the 24-gene signature to classify activities of *ESR1* fusions in the original training set. A cutoff of 0.3283 for mean signature score was defined.

**Supplementary Figure 5, related to Figure 5**


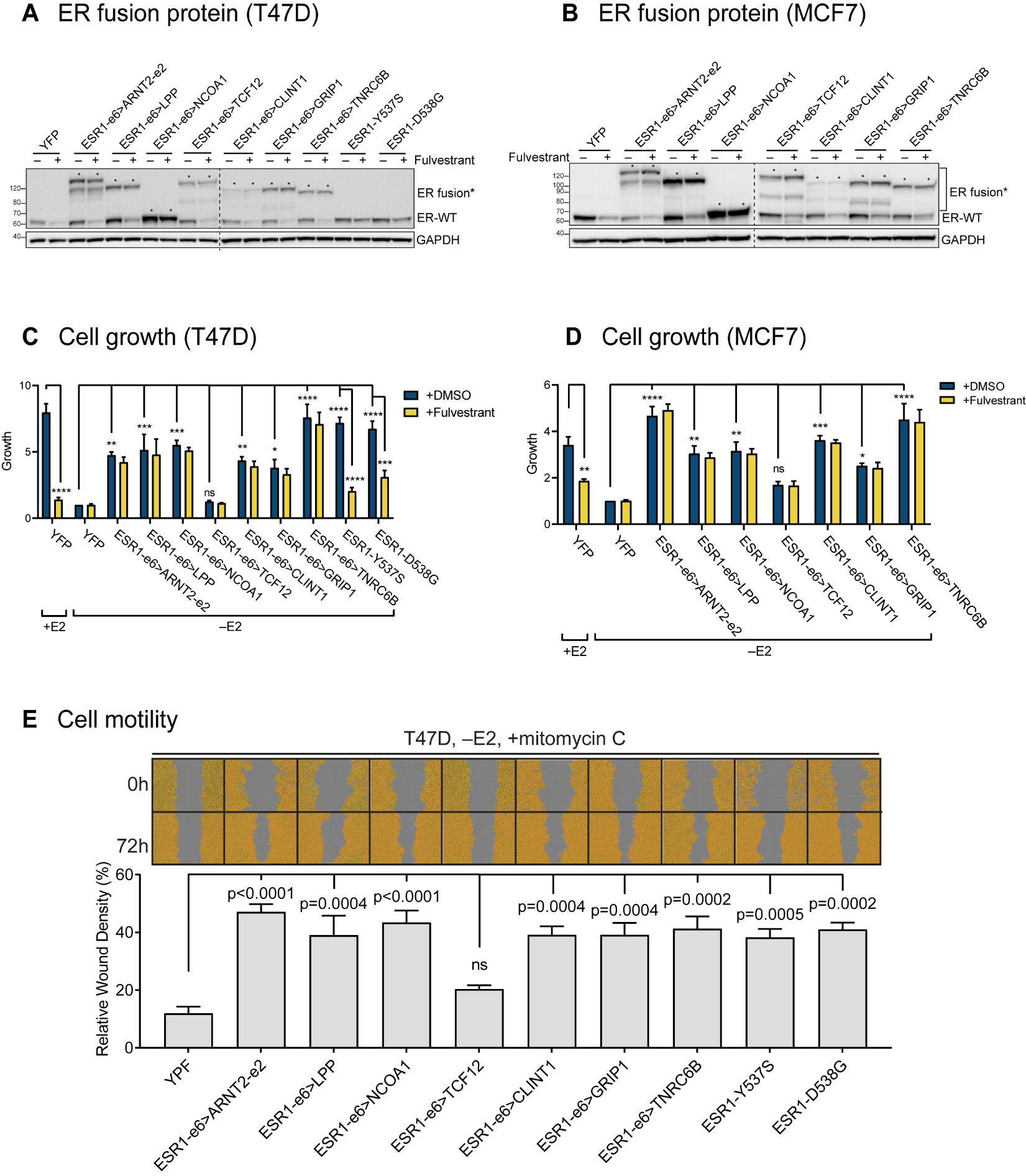


**Supplementary figure 5. Additional ESR1 fusions identified in ER+ MBC patients drive ET-resistant growth and promote hormone-independent motility of ER+ breast cancer cells. (A and B)** Immunoblotting of ERα and ESR1 fusion proteins with an N-terminal ERα antibody in hormone-deprived stably expressing T47D and MCF7 cells. Asterisks indicate ESR1 fusion proteins. GAPDH serves as a loading control. The dashed line indicates two separate blots that were conducted at the same time. The representative image is from 2-3 independent experiments. **(C and D)** Cell growth was assayed in hormone-deprived cells stably expressing an *ESR1* construct (mean ± SEM, n=3). One-way ANOVA followed by Dunnett’s multiple comparisons test was used to compare data of hormone-deprived *ESR1* fusion expressing cells to YFP control cells in vehicle control (+DMSO) group. Two-way ANOVA followed by Bonferroni’s test was used for multiple comparisons for each stable cell line after 100 nM fulvestrant treatment in the presence or absence of 10 nM E2. *p<0.05, **p<0.01, ***p<0.001, ****p<0.0001. **(E)** Cell motility was detected using scratch wound assays in hormone-deprived T47D cells stably expressing an *ESR1* construct and treated with 50 ng/ml mitomycin C to block proliferation (mean ± SEM, n=3). One-way ANOVA followed by Dunnett’s multiple comparisons test was used to compare each stable T47D cell line to YFP control cells (ns: not significant). Cells are pseudo-colored orange to aid visualization.

**Supplementary Figure 6, related to Figure 5**

**
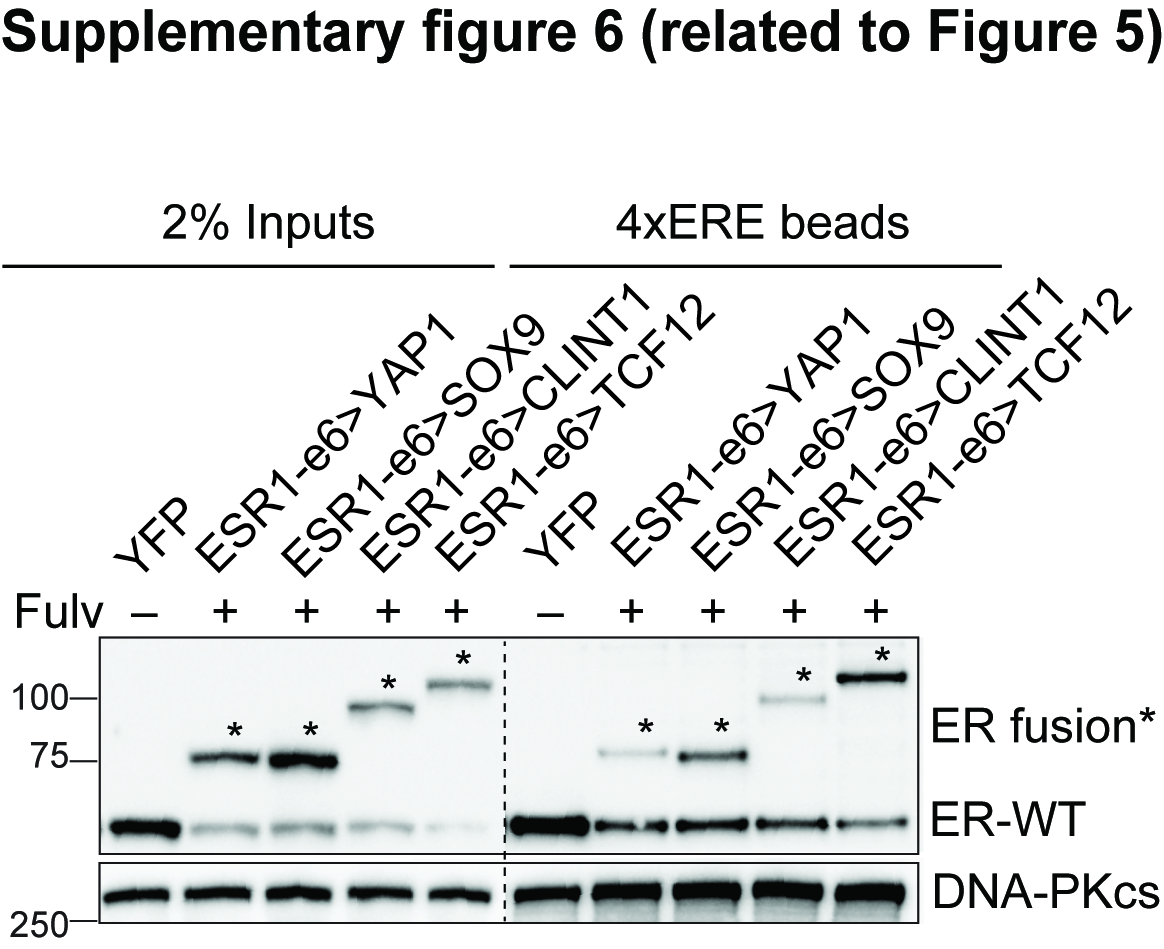
**

**Supplementary figure 6. Inactive ESR1-TCF12 fusion protein binds estrogen response element (ERE)-containing DNA like active *ESR1* fusion proteins.** Nuclear extracts of T47D cell lines expressing YFP, ESR1-e6>YAP1, ESR1-e6>SOX9, ESR1-e6>CLINT1, or ESR1-e6>TCF12 were prepared as described in the Methods section. All *ESR1* fusion-expressing cells were treated with 100 nM fulvestrant (Fulv) overnight to reduce the endogenous ERα level and thus its competition for binding to the EREs. ERE DNA pulldowns were done as described in the Methods section. *Top panel*, Immunoblotting of ERα and *ESR1* fusion proteins with an N-terminal ERα antibody reveals levels in 2% of starting inputs (*left*) and what was bound to 30% of the final 4xERE DNA beads (*right*). Asterisks indicate ER fusion proteins. *Bottom panel*, Immunoblotting of DNA-PK catalytic subunit (DNA-PKcs) serves as a loading control. The dashed line indicates two separate blots that were conducted at the same time.

**3) Supplementary Table**

**Supplementary table 1. Comprehensive summary of *ESR1* gene fusions in ER+ breast cancer**

| ***ESR1* fusions** | **Frame** | **Examples** | **Source** | **Mechanisms** |
| --- | --- | --- | --- | --- |
| Active fusions | In-frame | ESR1-e6>YAP1 ([4](#_ENREF_4),[5](#_ENREF_5))  ESR1-e6>PCDH11X (5)  ESR1-e6>SOX9 ([6](#_ENREF_6))  ESR1-e6>ARNT2-e18*  ESR1-e6>ARNT2-e2 ([7](#_ENREF_7))  ESR1-e6>LPP ([7](#_ENREF_7))  ESR1-e6>NCOA1 ([7](#_ENREF_7))  ESR1-e6>CLINT1 ([7](#_ENREF_7))  ESR1-e6>GRIP1 ([7](#_ENREF_7))  ESR1-e6>TNRC6B ([7](#_ENREF_7)) | Metastatic tumors | Generated by inter-chromosomal translocation  Produce stable ERα fusion protein  Upregulate transcriptional activation of ERα target and EMT genes  Drive ET-resistant tumor growth and metastasis |
|  | 5’UTR-CDS | ESR1-e2>CCDC170 ([8](#_ENREF_8))  ESR1-e2>C6orf211 ([9](#_ENREF_9)) | Primary tumors | Generated by tandem duplication  Produce truncated partner protein (rather than a chimeric protein)  Reduce ET sensitivity |
| Cell context-dependent fusion | In-frame | ESR1-e6>DAB2 ([6](#_ENREF_6)) | Metastatic tumors | Generated by inter-chromosomal translocation  Produce stable ERα fusion protein  Drive hormone-independent growth in MCF7 but not T47D cells |
| Inactive fusions | In-frame | ESR1-e6>GYG1 ([6](#_ENREF_6))  ESR1-e6>PCMT1*  ESR1-e6>ARID1B*  ESR1-e6>TCF12 ([7](#_ENREF_7)) | Metastatic tumors | Generated by inter-, or intra-chromosomal translocation  Produce stable ERα fusion protein  Do not drive E2-independent growth |
|  |  | ESR1-e6>NOP2 ([5](#_ENREF_5))  ESR1-e7>POLH ([5](#_ENREF_5))  ESR1-e6>AKAP12 ([5](#_ENREF_5),[9](#_ENREF_9)) | Primary tumors | Generated by inter-chromosomal translocation or tandem duplication  Do not drive E2-independent growth |
|  | Out-of-frame | ESR1-e3>CCDC170 ([5](#_ENREF_5))  ESR1-e4>CCDC170 ([5](#_ENREF_5))  ESR1-e5>CCDC170 ([5](#_ENREF_5))  ESR1-e6>AKR1D1 ([5](#_ENREF_5)) |  |  |

*Personal communication, Dr. Dan Robinson

**4) Supplementary References**

1. Peters TL, Kumar V, Polikepahad S, Lin FY, Sarabia SF, Liang Y*, et al.* BCOR-CCNB3 fusions are frequent in undifferentiated sarcomas of male children. Mod Pathol **2015**;28:575-86

2. Robinson MD, McCarthy DJ, Smyth GK. edgeR: a Bioconductor package for differential expression analysis of digital gene expression data. Bioinformatics **2010**;26:139-40

3. Rokita JL, Rathi KS, Cardenas MF, Upton KA, Jayaseelan J, Cross KL*, et al.* Genomic Profiling of Childhood Tumor Patient-Derived Xenograft Models to Enable Rational Clinical Trial Design. Cell Rep **2019**;29:1675-89 e9

4. Li S, Shen D, Shao J, Crowder R, Liu W, Prat A*, et al.* Endocrine-therapy-resistant ESR1 variants revealed by genomic characterization of breast-cancer-derived xenografts. Cell Rep **2013**;4:1116-30

5. Lei JT, Shao J, Zhang J, Iglesia M, Chan DW, Cao J*, et al.* Functional Annotation of ESR1 Gene Fusions in Estrogen Receptor-Positive Breast Cancer. Cell Rep **2018**;24:1434-44 e7

6. Hartmaier RJ, Trabucco SE, Priedigkeit N, Chung JH, Parachoniak CA, Vanden Borre P*, et al.* Recurrent hyperactive ESR1 fusion proteins in endocrine therapy-resistant breast cancer. Ann Oncol **2018**;29:872-80

7. Priestley P, Baber J, Lolkema MP, Steeghs N, de Bruijn E, Shale C*, et al.* Pan-cancer whole-genome analyses of metastatic solid tumours. Nature **2019**;575:210-6

8. Veeraraghavan J, Tan Y, Cao XX, Kim JA, Wang X, Chamness GC*, et al.* Recurrent ESR1-CCDC170 rearrangements in an aggressive subset of oestrogen receptor-positive breast cancers. Nat Commun **2014**;5:4577

9. Giltnane JM, Hutchinson KE, Stricker TP, Formisano L, Young CD, Estrada MV*, et al.* Genomic profiling of ER(+) breast cancers after short-term estrogen suppression reveals alterations associated with endocrine resistance. Sci Transl Med **2017**;9
